# Functional redundancy driven by isocitrate dehydrogenase enhances metabolic robustness in *Pseudomonas putida* KT2440

**DOI:** 10.1186/s12934-026-03053-3

**Published:** 2026-07-16

**Authors:** Maria-Tsampika Manoli, Sandra Herrera Alarcon, Álvaro Gargantilla-Becerra, M. Auxiliadora Prieto, Juan Nogales

**Affiliations:** 1 Polymer Biotechnology Group, Department of Biotechnology, Biological Research Centre Margarita Salas (CIB-CSIC), Madrid, Spain; 2 Interdisciplinary Platform for Sustainable Plastics Towards a Circular Economy-Spanish National Research Council (SusPlast-CSIC), Madrid, Spain; 3 Systems Biotechnology Group (SBG), Department of Systems Biology, Centro Nacional de Biotecnología (CNB-CSIC), Madrid, Spain; 4 CNB DNA Biofoundry (CNBio), CSIC, National Centre for Biotechnology (CNB-CSIC), Madrid, Spain; 5 Present Address: Tissue and Organ Homeostasis Scientific Program, CBM Severo Ochoa-CSIC, Madrid, Spain

**Keywords:** Isocitrate dehydrogenase, *Pseudomonas putida*, Metabolism, Robustness, Isoenzymes, Flux control

## Abstract

**Background:**

Prokaryotic isocitrate dehydrogenase (PIDH) is a conserved and highly versatile enzyme regulating carbon flux between the TCA cycle and the glyoxylate shunt. While most mesophilic organisms encode a single PIDH, the industrial chassis *Pseudomonas putida* KT2440 harbours two isoforms: a monomeric (IDH) and a dimeric (ICD). The physiological significance of this dual-system in mesophiles remains poorly understood, yet it may provide a framework for understanding metabolic flexibility and flux regulation in biotechnological applications.

**Results:**

Here, we biochemically characterized both PIDHs and, through modelling and site-directed mutagenesis, identified key residues (Ser133, Asn136, and Arg140) critical for monomeric catalytic activity and substrate binding. Genetic analysis revealed that the monomeric IDH is essential for growth, whereas the dimeric ICD appears to facilitate high-flux oxidative metabolism. System-level analysis integrating transcriptomics and flux modelling further demonstrated that these isoforms might fuel distinct TCA functional modes. Our results suggest that IDH sustains a basal, carbon-independent mode critical for biosynthetic precursor supply. In contrast, ICD drives a high-flux oxidative mode under glycolytic conditions and is post-translationally inactivated under gluconeogenic conditions, favouring the glyoxylate cycle.

**Conclusions:**

Taken together, these findings support a model in which specialized isoenzymes with conditional redundancy, rather than simple redundancy, in *P. putida*. This dual-enzyme system enables the cell to balance energy production with biosynthetic demands, which might facilitate metabolic adaptation under fluctuating environmental conditions. Our results provide a potential modular framework for engineering central metabolism to optimize precursor provisioning in microbial cell factories.

**Supplementary Information:**

The online version contains supplementary material available at https://doi.org/10.1186/s12934-026-03053-3.

## Introduction

Maintaining metabolic homeostasis and robustness requires a precise balance between energy generation and biosynthetic precursor supply. Functional redundancy can be defined as the existence of more than one similar or identical mechanisms that can replace each other when needed (e.g., response to environmental perturbations), thus ensuring system integrity by providing alternative mechanisms of robustness [1]. In this context, an in silico analysis using the genome scale metabolic model *i*JN1411 of *P. putida* revealed that up to 75% of non-essential genes contribute to robustness under genetic perturbations via alternative functional mechanisms [2, 3]. Notably, 59% of non-essential genes encode isoenzymes, highlighting the role of overlapping functions and enzymatic diversity as key drivers for maintaining metabolic stability [2, 3].

**Fig. 1:**
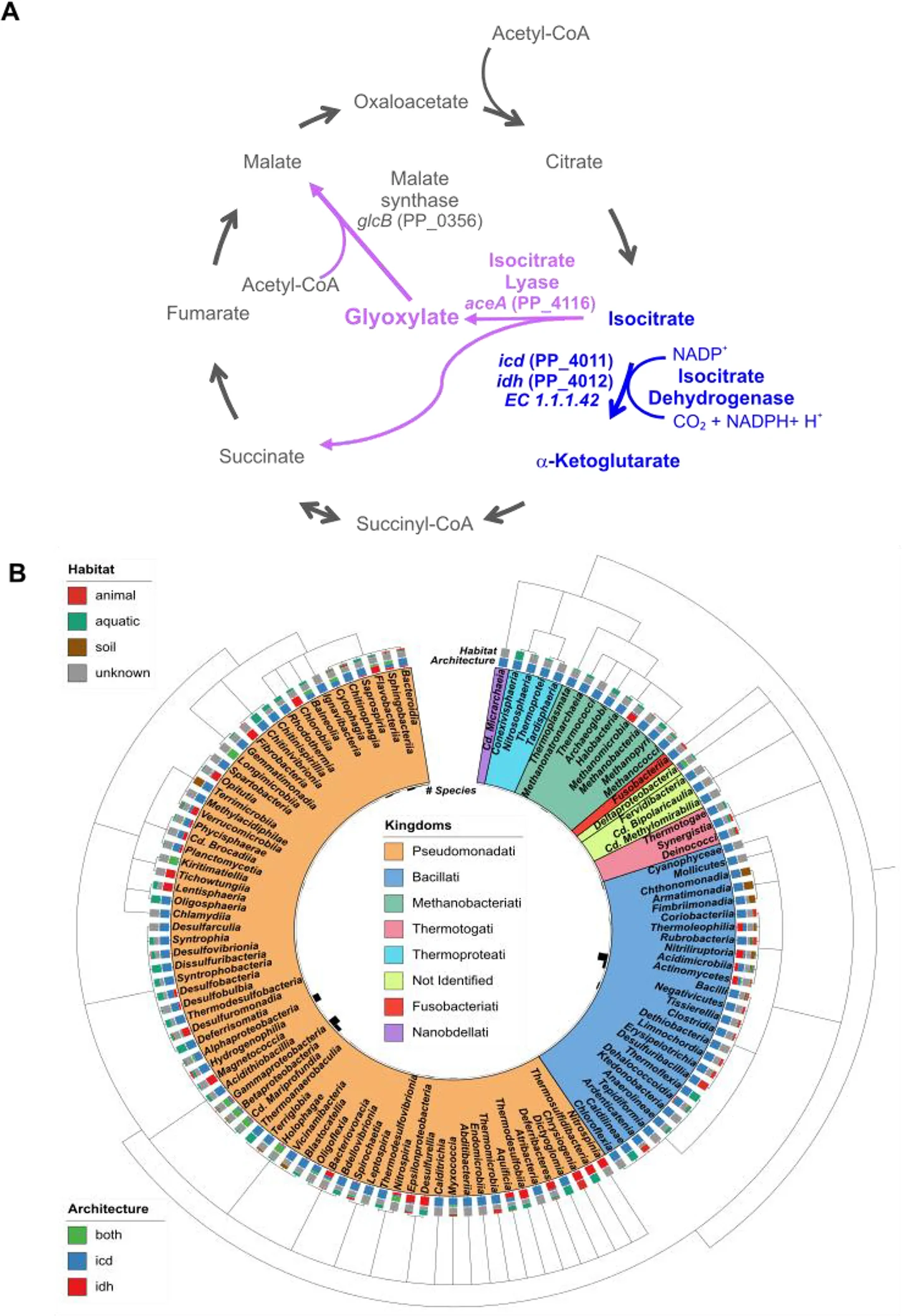
**A** A schematic representation of the tricarboxylic acid cycle (TCA) (gray) and its bifurcation into glyoxylate shunt (purple). Key enzymes of *P. putida*, isocitrate lyase (*aceA*), malate synthase (*glcB*) and isocitrate dehydrogenase (dark blue) are indicated. **B** Class level phylogenetic tree summarizing findings of IDH architecture. The resulting tree was formatted in Newick format and annotated with three composition datasets for visualization in Interactive Tree of Life (iTOL): (1) architecture composition, depicting the proportion of monomeric, dimeric, and mixed IDH variants per class; (2) habitat composition, showing the distribution of habitat types across classes according to BacDive database; and (3) kingdom composition, illustrating the phylogenetic breadth of the class-level sampling. Additionally, an abundance metric reflecting the total number of taxa within each class was generated to provide context for the representativeness of class-level observations

Isocitrate dehydrogenase is a pivotal enzyme in cellular metabolism, catalysing the oxidative decarboxylation of isocitrate to α-ketoglutarate and CO_2_ in the tricarboxylic acid (TCA) cycle [4, 5] (Fig. 1A). This reaction not only drives production of energy and key biomass building blocks such as α-ketoglutarate, but also generates reducing equivalents in the form of NADPH. These are essential for biomass building blocks, including amino acid, fatty acid, and steroid biosynthesis, as well as for cellular responses to oxidative stress [5–7].

Evolutionary studies indicate that the eubacterial NADP^+^-Isocitrate dehydrogenase (EC 1.1.1.42) evolved from the ancestral NAD^+^-Isocitrate dehydrogenase (EC 1.1.1.41) about 3.5 billion years ago, likely as an adaptive response to changing metabolic demands [4]. Most prokaryotic IDHs (PIDHs) are NADP^+^-dependent, and their activity is tightly linked to cellular demands for NADPH [4]. PIDHs exhibit significant structural diversity, existing either as monomeric or dimeric enzymes with distinct biochemical and regulatory properties. Monomeric isoforms (IDH), such as those found in *Azotobacter vinelandii*, are large single polypeptides (~ 80 to 100 kDa), whereas dimeric isoforms (ICD), exemplified by *Escherichia coli*, consist of two identical subunits (~ 40 to 50 kDa each) [8, 9]. Despite sharing conserved catalytic residues and evolutionary origins, their active sites are structurally distinct, with dimeric being regulated by phosphorylation via AceK kinase/phosphatase, whereas monomeric IDH lack this control. The monomeric enzyme contains a duplicated domain motif reminiscent of the dimeric interface, forming a catalytic site analogous to that of the dimeric enzyme. Despite this similarity, monomeric and dimeric PIDHs are not structurally homologous and are thought to have convergently evolved from distinct ancestral genes to possibly fulfil distinct physiological roles [10, 11].

Most bacteria encode a single type of PIDH, either monomeric or dimeric (Fig. 1 and Table S1). However, certain psychrophilic bacteria, such as *Colwellia maris and C. psychrerythraea,* harbor both isoenzymes [12–14]. The presence of different PIDH types has been associated with distinct biochemical properties and adaptive responses to environmental conditions, including low temperature, acetate availability, and other stress factors. For instance, the IDH in many psychrophilic organism functions as a cold-inducible gene, playing a critical role in survival under low-temperatures conditions and contributing to the metabolic robustness [15, 16]. Interestingly, several mesophilic species, including *P. putida* KT2440 [17], *P. aeruginosa* PAO1 [11], and *P. nautica* [18], also possess both PIDHs types as does the psychrophile *P. psychrophila* [14]*.* However, the physiological significance of this apparent redundancy in mesophiles remains poorly understood. Overall, comparative structural and kinetic analyses have revealed marked differences in substrate affinity, catalytic efficiency, and regulatory mechanisms between these two isoforms. The co-existence of monomeric and dimeric PIDHs in bacteria such as *P. putida* raises compelling questions regarding their evolutionary significance and functional specialization. Yet, the physiological roles of dual PIDHs in microbial systems remain poorly characterized, particularly in relation to their contributions to metabolic flexibility and stress resilience.

This study aims to elucidate the functional roles of PIDHs within the central metabolism of *P. putida* and to advance our understanding of microbial network architecture and evolutionary strategies for robustness. We demonstrate that both *icd* and *idh* genes are transcriptionally and enzymatically active in *P. putida*, and suggest the essentiality of *idh* for bacterial growth. Finally, we propose a “rheostatic” control mechanism that dynamically might regulate distinct operational modes of the TCA cycle driven by PIDHs, extending beyond the classical carbon partitioning between the TCA cycle and the glyoxylate shunt. These findings support the concept that specialized isoenzymes with conditional redundancy may contribute to flexible flux control through the TCA cycle in *P. putida*.

## Results and discussion

### *Pseudomonas putida* poses two transcriptionally active isocitrate dehydrogenases

*Pseudomonas putida* KT2440 encodes two PIDHs, a feature widely distributed among bacteria. The genomic organization of these genes is diverse across species. In psychrophilic bacteria such as *C. maris* and *C. psychrerythraea* the two PIDH genes are arranged in tandem with a conserved intergenic region and are independently regulated by distinct promoters (Fig. S1) [12–14].

In contrast, in *P. putida* KT2440, the dimeric (ICD, PP_4011) encoded by *icd* gene and monomeric (IDH, PP_4012) encoded by *idh* gene are located in tandem on the chromosome but in opposite orientations (Fig. S1) [19, 20]. This genomic arrangement, including a conserved intergenic region of 300–450 bps, is consistently conserved across pseudomonads, suggesting an evolutionary conservation of PIDH regulation and function [11, 14].

To assess the distribution of the two PIDHs architectures across bacteria, we analysed a representative dataset from ProGenomes4 comprising > 3,200 non-redundant bacterial and archaeal genomes. Sequences were processed into genome-aware datasets and partitioned into HMMER-compatible subsets for large-scale annotation. Across the dataset, genes encoding monomeric IDH, dimeric ICD, or both were widely distributed across bacterial phyla (Fig. 1B). However, most lineages preferentially encoded either IDH or ICD, suggesting that single-enzyme configurations represent the dominant evolutionary strategy. Notably, the co-occurrence of both isoforms was restricted to a limited number of phylogenetic groups and was particularly enriched within the Pseudomonadota, including Gamma-proteobacteria, the class that encompasses *P. putida*. Overall, these results indicate that dual-IDH/ICD architectures represent a lineage-restricted trait rather than a widespread bacterial strategy.

**Fig. 2:**
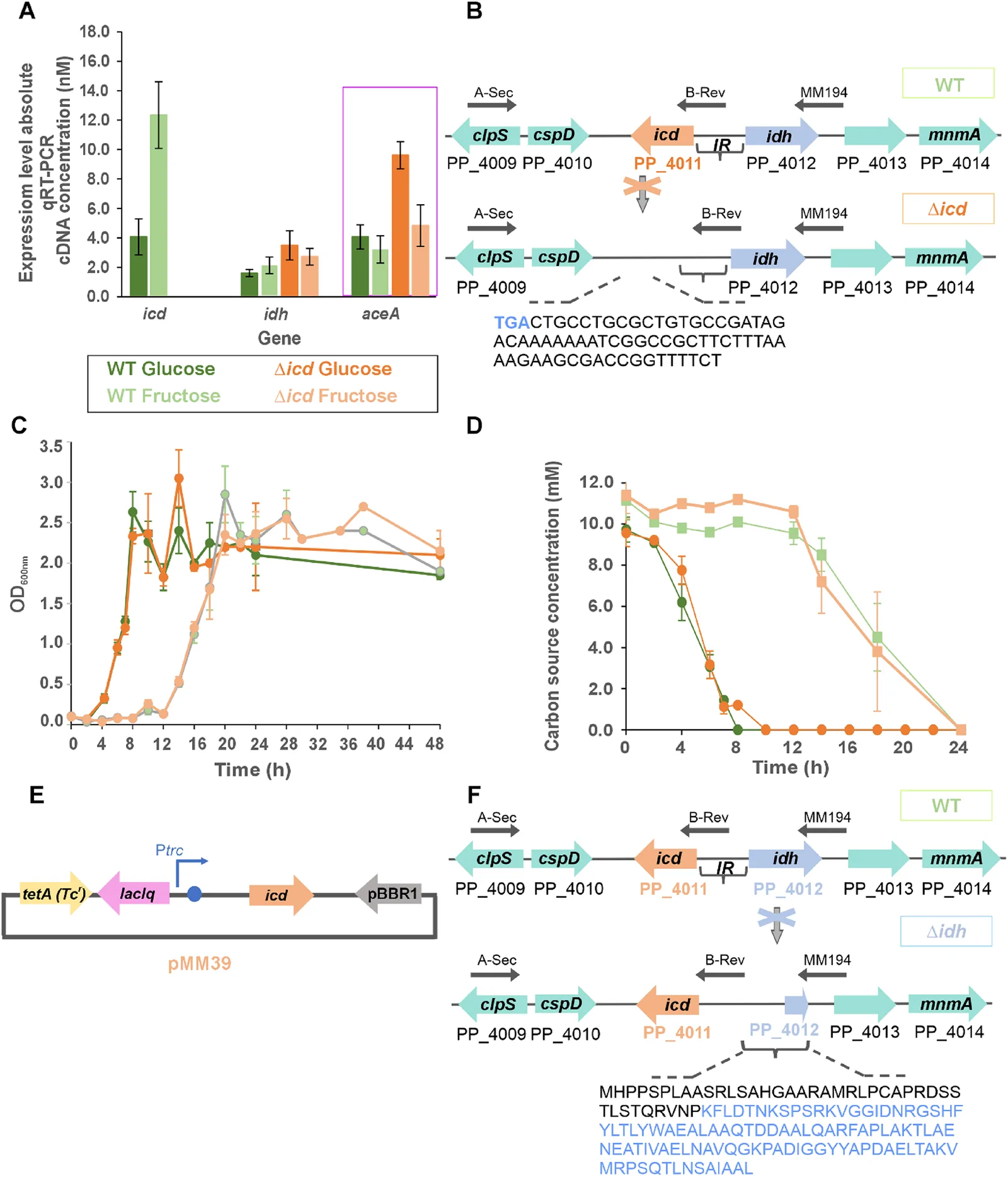
Phenotypic evaluation of isocitrate dehydrogenase deletion mutants. **A** qRT-PCR analysis of the transcription levels for PP_4011 (*icd* gene), PP_4012 (*idh* gene), and PP_4116 (*aceA* gene). These data represent the mean and standard deviation from two independent biological replicates. Comparison of KT2440/wt (green color) and Δ*icd*/PP_4011 (orange) strains in M63 minimal medium supplemented with 10 mM glucose (intense color) and fructose (light color). **B** Schematic representation of the *icd* deletion, showing remaining nucleotides post-deletion, in blue the remaining PP_4011 nucleotides. **C** Growth curves of the above-mentioned conditions; **D** Carbon consumption. **E** Schematic representation of the pIZ3 derived plasmid with *icd* overexpression (pMM39). **F** Schematic representation of the *idh* deletion, showing remaining amino acids post-deletion, in blue the remaining PP_4012 amino acid

To further investigate the two PIDHs in *P. putida*, we performed a transcriptional analysis on wild-type strain grown on glucose and fructose during mid-exponential phase (Fig. 2A). We found that *icd* was 3.5-fold higher under fructose growth compared to glucose, whereas *idh* maintained similar basal expression levels across both conditions (Fig. 2A). These findings confirm that both *icd* and *idh* are transcriptionally active in *P. putida* KT2440 under glucose and fructose growth. Additionally, *icd* expression appears to be carbon source dependent, while *idh* maintains a basal expression profile. This suggests that IDH likely acts as a dosage-limiting factor, compensating for the absence of *icd* and maintaining metabolic balance (see below).

### PIDHs in *P. putida* display a different profile of essentiality

To further investigate the roles of the *icd* and *idh*, we conducted a single and double deletion mutant analyses. Using the double recombination pK18*mobsacB* system, the *icd* was successfully deleted, generating the Δ*icd*/PP_4011 strain (Fig. 2B). When the growth performance of this strain was thoroughly analysed, based on OD, viable cell counts, and substrate consumption profiles using glucose and fructose as sole carbon sources, we found no significant differences compared to the wild-type strain (Fig. 2C, D). These results suggest the presence of certain functional redundancy, where the IDH likely compensates for the absence of ICD. Regarding the deletion of *idh*, despite numerous attempts employing various systems, including pK18*mob,* pK18*mobsacB,* and I-Sce-I, the *idh* gene could not be deleted. Efforts to create a double knockout strain also failed. Similar challenges were reported in *M. tuberculosis*, where the monomeric was deemed essential [21].

To overcome this limitation, the *icd* gene was cloned and overexpressed using a heterologous inducible promoter *Ptrc* in the wild-type strain background (i.e. harbouring both native *icd* and *idh* genes), resulting the strain KT2440 (pMM39) (Fig. 2E). Subsequently, we attempted the deletion of *idh* in this strain using the pK18*mobsacB* system (Fig. 2F). Second recombination plates were supplemented with α-ketoglutarate and glutamic acid to address potential emergent auxotrophy issues. Three independent clones with a potential *idh* null phenotype were obtained. PCR sequencing confirmed the deletion, although a 309 bp sequence remnant of *idh* remained in the genome (locus 4524069-4524377), resulting in a putative in-frame protein of 142 amino acids, including the C-terminal of IDH (Fig. 2F). While we cannot rule out that this genetic remnant preserves essential regulatory elements or promoter functions for downstream genes, its lack of the critical catalytic residues (Ser133, Asn136, Arg140) confirms a loss of IDH enzymatic function. In light of these results, and although monomeric IDH had not previously been classified as conditionally essential in *P. aeruginosa* or *P. putida* growth [22–25], our findings indicate that the catalytic activity of IDH is conditionally required for optimal growth under native expression levels. The ability to delete *idh* only upon ectopic *icd* overexpression represents a classic “bypass of essentiality” where increasing the dosage of a specialized paralog compensates for the loss of a required metabolic node. In line with this hypothesis phenotypic analysis of the IDH-null strain ΔIDH/PP_4012 (pMM39) under various growth conditions, including LB, LB with 15 mM α-ketoglutarate, M63 minimal medium with 15 mM α-ketoglutarate, or 10 mM glucose, showed no major growth differences compared to the wild type (Fig. S3). These observations indicate that the native ICD, along with its overexpression, can compensate for the absence of IDH in a large array of nutritional scenarios, highlighting conditional functional compensation between the two isoforms. However, under normal physiological conditions without ICD overexpression, IDH appears indispensable, underscoring its critical role in maintaining metabolic balance in *P. putida* KT2440. Future physiological studies employing a broader range of non-glycolytic substrates, such as acetate or succinate, will be necessary to fully map the conditional requirements of this mutant across different metabolic regimes.

### Structural properties of isocitrate dehydrogenase isoenzymes

To better understand the physiological relevance of these enzymes, and their condition-dependent functional redundancy, we next investigated their structural and functional features including substrate and cofactor-binding properties, through sequence alignments and structural analyses (Fig. S4-S6). The ICD shares high sequence identity (75–91%) with orthologs from mesophilic and psychrophilic bacteria (e.g. *C. maris*, *E. coli*, *P. aeruginosa* PAO1, and *P. psychrophila*) while the IDH shows slightly lower identity (66–82%). Despite sharing only 8% identity with each other, these enzymes exhibit conserved residues essential for isocitrate and metal binding suggesting distinct evolutionary pathways but similar substrate recognition mechanisms [10, 26]. Yasutake and collaborators, suggested that monomeric IDH may have evolved through the duplication of the B-C domains from an ancestral dimeric ICD (Fig. S6A). Crystal data from *A. vinelandii* confirm that residues involved in isocitrate and metal binding are identical in both forms, though NADP^+^ interaction differs due to partial residue divergence [10, 26].

Based on ICD structural data, the residues conferring NADP^+^ specificity include Asp292′ (the prime sign indicates the second subunit), Lys344, Tyr345, Val351, Tyr391, and Arg395, with Lys344 and Tyr345 being key and conserved in *P. putida* (Fig. S2, Fig. S6A) [10, 27]. In contrast, IDH shows alternative NADP^+^ binding residues: Arg358, Lys588, His589, and Arg649, indicating distinct coenzyme recognition (Fig. S5-S6) [10, 27]. Predicted isocitrate binding residues are Ser113, Asn115, Arg129, Arg153, Tyr160, and Lys230’ for ICD, and Ser132, Asn135, Arg139, Arg145, Arg547, Tyr420, and Lys255 for IDH [10, 27]. Metal binding residues include Asp283´, Asp307, and Asp311 in ICD, and Asp350 and Asp548 in IDH.

Experimental data from psychrophilic bacteria identified Glu55 as a determinant of mesophilic stability in *P. psychrophila* IDH (Fig. S5) [14, 28, 29]. This residue, along with others conserved in *P. putida* KT2440, likely contributes to the mesophilic properties of its monomeric IDH.

### Functional characterization of purified isocitrate dehydrogenase enzymes

**Fig. 3:**
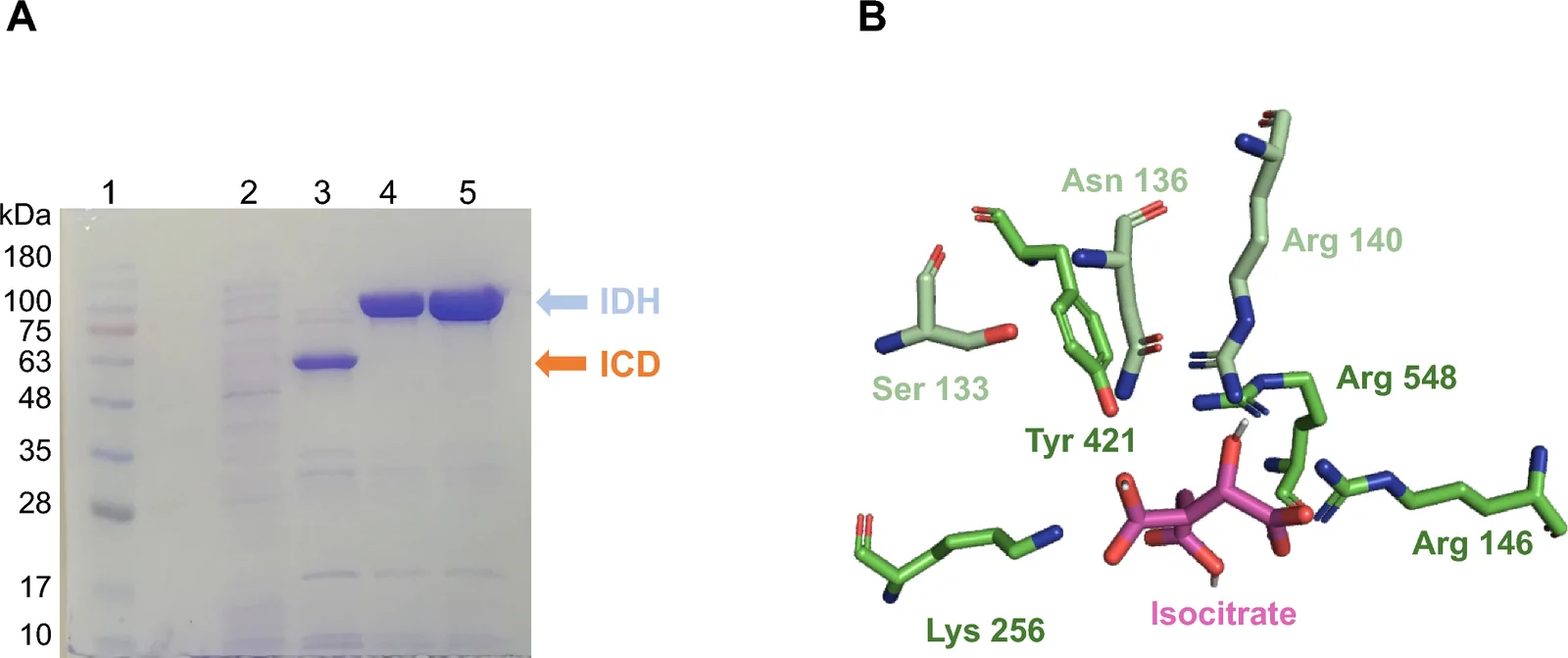
**A** SDS-PAGE analysis (12.5% polyacrylamide gel) of purified PIDH proteins using His-trap columns. Lanes: 1. Blue Star Prestained protein molecular weight marker, 2. Negative control (empty pET29a), 3. ICD, pMM27, 4. IDH, pMM129, 5.IDH/ Ser133Ala, Asn136Ala, Arg140Ala triple mutant, pMM42. ** B** A close-up view of the molecular docking result between isocitrate and conserved active site residues in IDH, residues mutated in this study are highlighted in light green, isocitrate in pink

To investigate the functional and kinetic properties of the PIDH isoenzymes in *P. putida* KT2440, both isoforms were heterologously overexpressed in *E. coli* BL21 (DE3, pLysS) (for strains info in Table 1) and purified via His-tag affinity chromatography (Material and methods section, Fig. 3A, Table 2). Both enzymes were catalytically active and showed no detectable activity with NAD^+^, confirming NADP^+^ as the preferred cofactor, consistent with the low Km values obtained for NADP^+^ (44.6 µM for ICD and 64.2 µM for IDH) similar to those reported for *P. aeruginosa* PIDHs (32.3–34.5 µM) [11]. Kinetic analyses revealed that IDH displayed a lower K_m_ for isocitrate (15.7 µM) than ICD (65.9 µM), indicating higher substrate affinity, in agreement with previous findings in *Corynebacterium glutamicum*, *Azotobacter vinelandii*, and *P. aeruginosa* [11, 27, 30].

**Table 1:** Strains and plasmids used in this study, including genotypes and key characteristics

Strains	Genotype/relevant characteristics	References
*P. putida*		
KT2440	TOL plasmid-cured, spontaneous restriction deficient derivative of *P. putida* mt-2, wild type	[54]
PP05_19	KT2440 derivative strain, Δ*icd*/PP_4011	This work
KT2440 (pMM39)	Tc^r^, KT2440 harboring pMM39 plasmid	This work
PP05_20 (pMM39)	Tc^r^, KT2440 harboring pMM39 plasmid with Δ*idh*/PP_4012 locus affected	This work
*E. coli*		
DH10B	Cloning host; F-, *mcrA* Δ(*mrr hsdRMS-mcrBC*) Φ80d*lac*ΔM15 Δ*lacX74 deoR recA1 araD139* Δ(*ara-leu*)7697	Invitrogen, Thermo Fisher Scientific, USA
HB101	Helper strain; F^−^ λ^−^ *hsdS20(r*_*B*_^*−*^* m*_*B*_^*−*^*) recA13 leuB6*(Am) *araC14* Δ(*gpt-proA*)62 *lacY1 galK2*(Oc) *xyl-5 mtl-1 rpsL20*(Sm^R^) *glnX44*(AS)	[55]
BL21 (λDE3, pLysS)	Cm^r^, B F^–^ *ompT gal dcm lon hsdS*_*B*_(*r*_*B*_^–^*m*_*B*_^–^) λ(DE3 [*lacI lacUV5*-*T7p07 ind1 sam7 nin5*]) [*malB*^+^]_K-12_(λ^S^)	Novagen, Merck KGaA, Germany
Plasmids		
pRK600	Cm^r^, ColE1 *oriV* RK2 Mob^+^ Tra^+^ donor of transfer functions	[56]
pK18*mobsacB*	Km^r^, ColE oriV, Mob^+^, lacZa, *sacB*; vector for allelic exchange homologous recombination mutagenesis	[57]
pET29a (+)	Km^r^, expression vector. F1 ori, T7 promoter/lac operator, N-terminal S·Tag™/thrombin configuration, and optional C-terminal His6-tag sequence	Novagen, Merck KGaA, Germany
pIZ2	Gm^r^, extended pIZ1016 polylinker. EcoRI, ClaI, ScaI, SmaI (XmaI), SpeI, XvaI, SalI, PstI (SbfI), SphI, HindIII, and SacI	[58]
pIZ3	Tc^r^, pIZ2 derived plasmid	This work, kindly provided by Dr. Gonzalo Durante CIB-CSIC
pMM19	Km^r^, pK18*mobsacB* derivative vector used for *icd* deletion	[35]
pMM27	Km^r^, pET29a (+) derivative vector for production of ICD-His_6_ tag sequence	This work
pMM129	Km^r^, pET29a (+) derivative vector for production of IDH-His_6_ tag sequence	This work
pMM130	Km^r^, pET29(+) derivative vector for production of IDH mut-His_6_ tag sequence, with point mutations to Asn136Ala	This work
pMM131	Km^r^, pET29(+) derivative vector for production of IDH mut-His_6_ tag sequence, with point mutations to Ser133Ala	This work
pMM42	Km^r^, pET29(+) derivative vector for production of IDH mut-His_6_ tag sequence, with point mutations to Ser133Ala, Asn136Ala, Arg140Ala	This work
pMM39	Tc^r^, pIZ3 derived bearing ICD wt	This work

**Table 2:** Kinetic parameters of dimeric ICD and monomeric IDH proteins, including wild type and mutant versions, measured in 50 mM Tris–HCl (pH 7.2)

Plasmid/dialyzed enzyme	Isocitrate				NADP^+^			
	*K*_*m*_ (µM)	V_max_ (ΔmM min^−1^)	*k*_*cat*_ (s^−1^)	*k*_*cat*_*/K*_*m*_ (s^−1^ µM^−1^)	*K*_*m*_ (µM)	*V*_*max*_ (ΔmM min^−1^)	*k*_*cat*_ (s^−1^)	k_cat_/*K*_*m*_ (s^−^1 µM^−1^)
pET29a (empty plasmid)	N.A	N.A	N.A	N.A	N.A	N.A	N.A	N.A
pMM27* (ICD)	65.9 ± 9.8	0.1 ± 0.01	28.0 ± 2.8	0.4 ± 0.07	44.6 ± 4.9	0.1 ± 0.00	24.7 ± 2.5	0.6 ± 0.09
pMM129 (IDH)	15.7 ± 1.4	0.5 ± 0.01	142.9 ± 2.9	9.1 ± 0.83	64.2 ± 7.2	0.5 ± 0.01	172.5 ± 3.5	2.7 ± 0.31
pMM131 (IDH/Ser133Ala)	59.6 ± 5.1	0.2 ± 0.00	56.0 ± 2.8	0.9 ± 0.09	138.2 ± 10.5	0.3 ± 0.01	71.7 ± 2.4	0.5 ± 0.04
pMM130 (IDH/Asn136Ala)	20.8 ± 2.8	0.2 ± 0.00	11.7 ± 0.6	0.6 ± 0.08	51.5 ± 9.6	0.2 ± 0.01	13.6 ± 0.7	0.3 ± 0.06
pMM42 (IDH/Ser133Ala, Asn136Ala, Arg140Ala)	N.A	N.A	N.A	N.A	N.A	N.A	N.A	N.A

K_m_ and V_max_ values were determined by fitting data to the Michaelis-Menten model in Graphpad Prism 6. K_cat_ values were calculated from V_max_. Standard deviations for K_cat_ and K_cat_/K_m_ were derived by applying standard mathematical error propagation to the variance of the independent variables Vmax and Km). N.A.: not applicable. Data are represented as the mean and standard deviation from three replicates. *Kinetic data for ICD were obtained from non-dialyzed samples in phosphate buffer due to enzyme instability post-dialysis.

Since both enzymes are biochemically active and *icd* could be deleted without affecting cell viability, but all attempts to delete *idh* failed, we focused on elucidating the biochemical basis of IDH function, hypothesizing a possible structural or non-enzymatic essential role. For this, site-directed mutagenesis targeted key residues predicted to mediate isocitrate binding and catalysis (residues Ser133, Asn136) (Fig. 3B). The single mutants Ser133Ala and Asn136Ala exhibited altered catalytic parameters (Table 2). Specifically, they showed increased K_m_ values for isocitrate (59.6 µM, 20.8 µM, respectively compared to 15.7 µM for the wild type) indicating a slightly diminished substrate affinity, while retaining a strong preference for NADP^+^. Single mutants (Ser133Ala, Asn136Ala) also showed reduced V_max_ (i.e. 0.2 vs 0.5 ΔmM·min^−1^), K_cat_ (i.e. 56 and 11.7 vs 142.9 s^−1^), and catalytic efficiency (K_cat_/K_m_; i.e. 0.9 and 0.6 vs 9.1 s^−1^·µM^−1^) compared to the wild type IDH, confirming these residues’ essential contributions to substrate binding and enzyme kinetics (Table 2). As an effort to find a loss of function mutant, we decided to mutate the three proposed amino acid for isocitrate binding (Fig. 3B). Notably, the triple mutant (Ser133Ala, Asn136Ala, Arg140Ala) completely lost enzymatic activity, underscoring the essential role of these residues for catalysis. Consistently, attempts to generate an *idh* deletion mutant in *P. putida* via double recombination using the inactive triple mutant allele were unsuccessful, as only wild-type genotypes were recovered. This genetic evidence argues against a putative structural or non-enzymatic role for IDH and instead further supports its essential enzymatic function in *P. putida* KT2440 viability.

Overall, we conclude that the two native PIDH isoenzymes in *P. putida* KT2440 are enzymatically active and exhibit a clear preference for NADP^+^ over NAD^+^. The two enzymes display distinct kinetic profiles, with IDH showing superior catalytic performance. Furthermore, we demonstrated that key residues Ser133, Asn136, and Arg140 in IDH are critical for catalytic function. Accordingly, the absence of this region in the IDH-null strain confirms the loss of enzymatic activity due to IDH in this strain.

### System level analysis of central metabolism in *P. putida* identify multiple TCA cycle functional modes fuelled by both PIDH

**Fig. 4:**
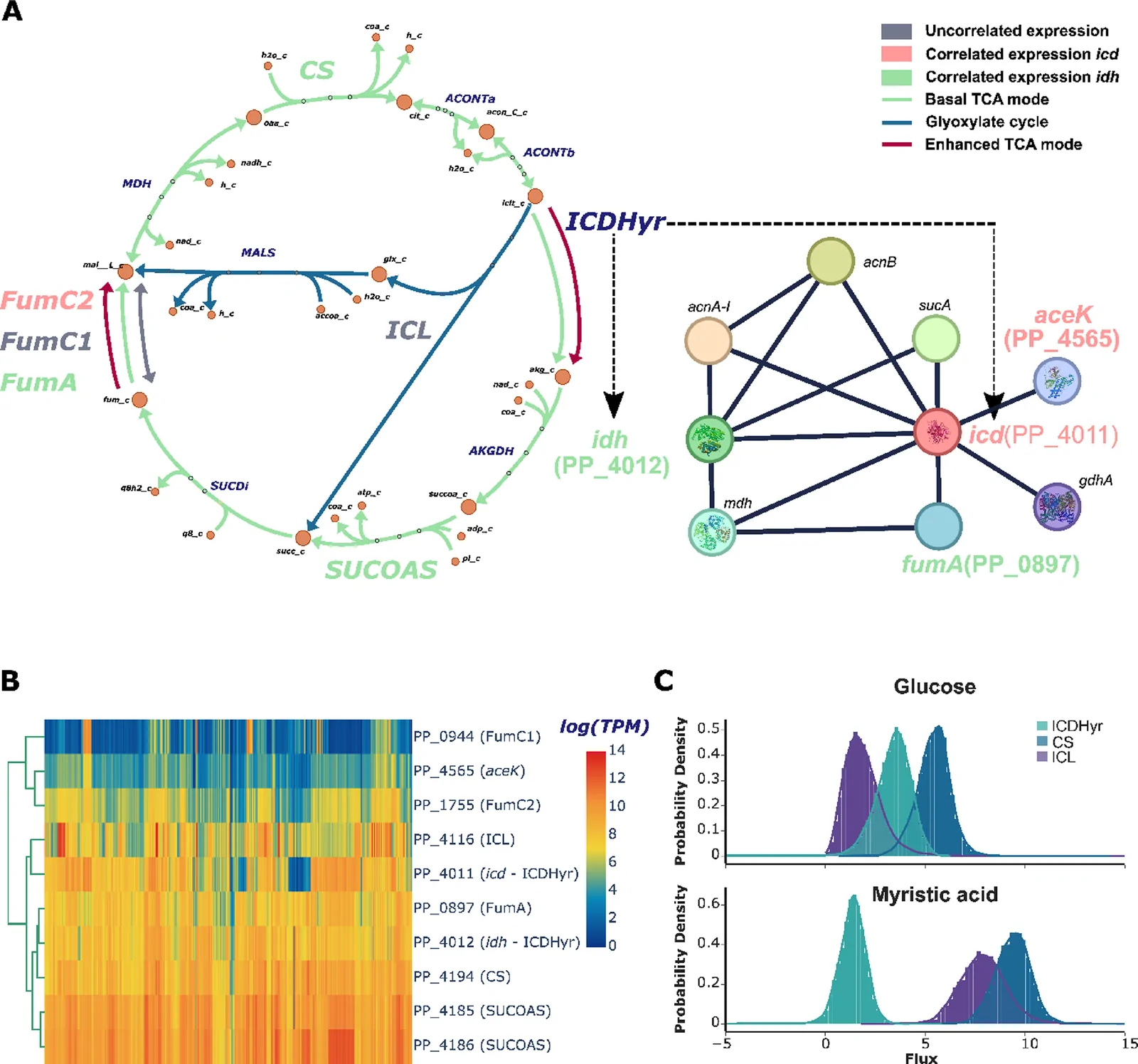
Analysis of the metabolic context of ICDHyr. **A** Graph representing TCA reactions surrounding ICDHyr (left) and the interaction graph of *idh* and *icd* coding proteins according to STRING (right). Reactions are represented as coloured arrows according to the proposed metabolic mode where they participate, while reaction labels are coloured depending on the expression correlation of the reaction gene(s) with *icd* (red) or *idh* (green). **B** Clustered heatmap of expression levels from each of the genes highlighted in the previous panel. Expression data was extracted from the putidaPRECISE321 dataset. Detailed pairwise correlation analyses are shown in Fig. S5. **C** Histogram plots displaying the results of a flux sampling analysis performed over 2 GEMs contextualized with expression data from *P. putida* growing in minimal media with glucose (top) or myristic acid (bottom) as the only carbon source. Note that each of reactions represents a metabolic mode mentioned in (**A**). Flux sampling was carried out with 10,000 samples per condition with a thinning parameter of 1,000 s

To clarify the functional context of the two isocitrate dehydrogenase isoforms (ICDHyr reaction) in *P. putida*, we first constructed a network-based representation of reactions surrounding ICDHyr using a STRING-derived interaction graph to identify the immediate metabolic neighbourhood of ICD and IDH in *P. putida*. We then analysed their transcriptional context of this subnetwork under different nutritional scenarios (Fig. 4A-B). For this, we leveraged the putidaPRECISE321 dataset (https://imodulondb.org/dataset.html?organism=p_putida&dataset=precise321), which provides standardized gene expression profiles across 321 conditions involving diverse carbon sources. In line with this systems-level framework, the STRING network and the associated clustering analyses represent associative relationships derived from large-scale transcriptomic datasets rather than direct regulatory interactions.

Specifically, *idh* expression showed strong correlation with *sucCD* (encoding the two subunits of succinyl-CoA synthetase), *fumA* (fumarase), and *gltA* (citrate synthase) across all conditions (Fig. S5A-D). This high expression correlation with key TCA enzymes is consistent with its potential role in a basal, carbon-independent TCA functional mode.

In contrast, *icd* expression showed no significant correlation with other genes involved in the TCA cycle but exhibited a strong positive correlation with *aceK*, which encodes the bifunctional isocitrate dehydrogenase kinase/phosphatase responsible for controlling flux partitioning between the TCA cycle and the glyoxylate shunt by modulating ICD activity. In Gram negative bacteria, AceK-mediated phosphorylation regulates ICD activity in a carbon-source dependent manner: ICD is predominantly active and dephosphorylated under glycolytic growth conditions, whereas under gluconeogenic conditions AceK-mediated phosphorylation leads to ICD inactivation and a redistribution of carbon flux towards the glyoxylate shunt. This regulatory mechanism has been well characterised in several species, including *P. aeruginosa* [11], a closely related species to *P. putida* ICD (Fig. S6). This indicates that the canonical AceK phosphorylation site (Ser115) is conserved, suggesting that ICD in *P. putida* may be subject to AceK-mediated post-translational regulation analogous to that described in related microorganisms. Taken together, these observations are compatible with a coordinated behaviour between *icd* and *aceK* across conditions.

Under glycolytic conditions such as glucose-rich media, *icd* expression is associated with an ioxidative TCA configuration, consistent with increased flux through the cycle. This interpretation is supported by the correlation of *icd* with alternative fumarase isoforms (*fumC2*) (Fig. 4A, B, Fig. S5), which may contribute to maintaining TCA flux capacity under conditions where FumA, the primary fumarase in *P. putida* becomes saturated. Conversely, under gluconeogenic conditions such as acetate or ethanol utilization, the regulatory paradigm described in other organisms predicts AceK-mediated inhibition of ICD activity, thereby favouring flux through the glyoxylate shunt. In this context, increased expression of isocitrate lyase (*aceA)* is observed, whereas, *icd* expression does not correlate with *aceA* (ICL) at the transcriptional level (Fig. S5). Nevertheless, the induction of *aceA* in the *icd* deletion strain Δ*icd*/PP_4011 (Fig. 2A) suggests a compensatory activation of the glyoxylate shunt when ICD function is absent. These findings are conceptually consistent with a “rheostatic control” mechanism previously described in *P. aeruginosa*, where ICD and ICL alternate between oxidative and biosynthetic replenishment flux modes depending on nutrient status [11]. In *P. putida*, our data support a similar systems-level organization, although direct experimental validation of AceK-mediated phosphorylation under the conditions tested remains to be demonstrated.

Overall, our results are consistent with the existence of three distinct functional TCA modes in *P. putida*, driven by the two isocitrate dehydrogenase isoforms: (i) a basal mode, largely independent of carbon source and primarily mediated by IDH; (ii) a carbon-dependent oxidative mode, prioritizing energy generation and driven by ICD; and (iii) a carbon-dependent glyoxylate mode, favouring the provisioning of biosynthetic precursor supply and associated with reduced ICD activity through post-transcriptional regulation. Among these three configurations, our data suggest that the basal mode mediated by IDH is the only configuration strictly required for growth.

### Carbon flux distribution suggests an essential role of IDH providing basal TCA activity

To further support this hypothesis, we investigated carbon fluxes through the isocitrate dehydrogenase (ICDHyr) hub using the latest genome-scale metabolic model of *P. putida* (*i*JN1462) [3]. For this, we constrained the model using transcriptomic data for growth on glucose, representing conditions associated with an oxidative TCA configuration and myristic acid (MA), which is catabolized to acetyl-CoA and is expected to rely more strongly on the glyoxylate shunt for precursor supply (Fig. 4B). We systematically compared the solution space of these two condition-specific models. Although flux through ICDHyr cannot be decoupled between ICD and IDH, the overall ICDHyr flux in the MA-specific model can be interpreted as primarily reflecting IDH-associated activity, assuming that under this condition ICD is phosphorylated as occurs in *P. aeruginosa*, thus favouring the glyoxylate-driven biosynthetic mode. In contrast, glucose conditions are associated with increased flux through ICDHyr consistent with an oxidative TCA configuration.

In good agreement with this hypothesis, we found a large flux through ICDHyr under glucose, accounting for a substantial fraction of the flux through citrate synthase (CS) (Fig. 4C). Conversely, only a small flux through ICDHyr, mainly reflecting the basal TCA mode, was observed when MA was used as the carbon source, with most of the flux through citrate synthase being redirected to the glyoxylate cycle via ICL (Fig. 4C).

Taken together, these results are consistent with a relevant role of IDH in maintaining basal TCA activity in *P. putida*, largely independent carbon source-dependent regulation. This activity is likely related to the precursor-provisioning role of the TCA cycle, supplying key biosynthetic building blocks such as 2-oxoglutarate rather than acting primarily as an energy driver. In contrast, ICD appears to function mainly as a driver of high flux through the TCA cycle under favourable conditions, providing both biosynthetic precursors and energy. These findings are fully consistent with the gene essentiality pattern of *idh*, but not of *icd*, reported above. From a systems biology perspective, this dual-isoform “rheostat” architecture identifies IDH as a stable metabolic anchor and ICD as a tuneable valve, and may provide a framework for future metabolic engineering carbon flux redirection in *P. putida* cell factories.

## Conclusion

This study unveils the dual isocitrate dehydrogenase system of *P. putida* as a finely tuned regulatory mechanism balancing energy production and biosynthetic demands. While most bacteria use a single NADP⁺-dependent IDH to control carbon flow through the tricarboxylic acid (TCA) cycle, *P. putida* and other members of bacteria classes belonging Pseudomonadota kingdom encodes two distinct enzymes: a monomeric IDH and a dimeric ICD. Taken together, the data presented in this study show that *P. putida* KT2440 relies on a dual isocitrate dehydrogenase system to achieve expanded metabolic flexibility and robustness. This behaviour is conceptually consistent with the broader genetic logic of bypassable essentiality, in which paralogous enzymes and pathway-level buffering mechanisms enable conditional redundancy, as described in other systems such as yest metabolic networks [31, 32].

From a biochemical standpoint, both isoforms were active NADP⁺-dependent dehydrogenases, yet they differ significantly in structure, kinetics, and physiological role. The monomeric IDH shows higher substrate affinity and catalytic efficiency, providing a stable baseline of TCA cycle activity. In contrast, the dimeric ICD exhibits lower affinity but is more dynamically regulated, acting as a metabolic “switch” that adjusts to environmental and nutritional cues. Mutagenesis of key residues (Ser133, Asn136, and Arg140) completely abolished IDH activity, confirming that its catalytic role is essential for cell viability.

Genetic analysis revealed that *idh* could not be deleted under normal conditions, highlighting its indispensability, whereas the *icd* gene could be removed without affecting growth. Together, these observations support a model of specialised isoenzymes with conditional redundancy, in which both PIDHs fulfil distinct physiological roles while retaining a partial capacity for functional compensation under specific genetic contexts. This indicates that the monomeric enzyme sustains fundamental metabolic fluxes, while the dimeric form adds flexibility rather than redundancy. Transcriptomic and metabolic modelling further supported this division of labour: IDH maintains a constant, carbon-independent TCA mode that guarantees biosynthetic precursor supply, whereas ICD drives an oxidative, energy-producing mode under glycolytic conditions. When nutrients are scarce or carbon sources are gluconeogenic, ICD becomes inactivated, redirecting carbon toward the glyoxylate shunt to preserve resources.

Taken together, these findings illustrate how *P. putida* uses enzyme diversity as an adaptive tool. Instead of simple genetic backup, the coexistence of monomeric and dimeric IDHs enables the bacterium to fine-tune its metabolism in real time, balancing energy generation with biosynthetic needs. This dual system may function as a metabolic rheostat-like architecture, potentially contributing to metabolic robustness against environmental fluctuations and genetic stress. From this perspective, the system represents a bacterial example of a broader principle of bypassable essentiality at central metabolic nodes. Beyond its biological significance, understanding this mechanism opens new perspectives for metabolic engineering, providing a mechanistic blueprint that can inform the design of more resilient microbial platforms for biotechnological production and potentially, identifying new enzymatic targets for antimicrobial development.

## Material and methods

### Bacterial strains, media and growth conditions

*E. coli* and *P. putida* strains were routinely cultured for DNA manipulations and pre-cultures in lysogeny broth (LB) medium at 37 °C and 30 °C, respectively [33, 34]. Antibiotics and reagents were added as required: chloramphenicol (34 µg/mL), ampicillin (100 µg/mL), kanamycin (50 µg/mL), tetracycline (5 µg/mL), IPTG (1 mM), and Xgal (40 µg/mL) were added where needed. Bacterial strains and plasmids are detailed in Table 1.

Standard growth experiments for *P. putida* were performed in M63 minimal medium (13.6 g of KH_2_PO_4_/L, 2 g (NH_4_)_2_SO_4_/L, 0.5 mg FeSO_4_·7H_2_O/L, adjusted to pH 7.0 with KOH), plus the corresponding carbon source needed. The medium was supplemented with 1 mM MgSO_4_ and a solution of trace elements (Goodies) (composition 1000X dissolved in 1N HCl: 2.78 g/L of FeSO_4_·7H_2_O, 1.98 g/L of MnCl_2_·4H_2_O, 2.81 g/L of CoSO_4_·7H_2_O, 1.47 g/L CaCl_2_·2H_2_O, 0.17 g/L of CuCl_2_·2H_2_O, 0.29 g/L of ZnSO_4_·7H_2_O) [35].

LB preculture cells were washed twice with 0.85% saline solution, adjusted to an OD_600_ of 0.1 and cultured in M63 medium under shaking at 200 rpm in 100 ml Erlenmeyer flasks at 30 °C. Growth was monitored spectrophotometrically at 600 nm for 24 h [34].

### Construction of *P. putida* KT2440 *icd* deletion mutant

The *icd* (PP_4011) gene was deleted using the pK18*mobsacB* system [35–38]. Homology arms (~ 500 bp upstream and downstream of PP_4011) were amplified with primers containing HindIII-HF and BamHI-HF restriction sites (Table S2). The fragment was cloned into pK18*mobsacB* and verified by sequencing. The construct was introduced into *P. putida* KT2440 genome via triparental filter mating [39]. First recombination events were selected on cetrimide agar with kanamycin and recombinants were confirmed by PCR [37, 40]. For the second recombination, strains were grown in LB without antibiotic during 6 h and plated on M63 minimal medium with 5% sucrose and 5 mM sodium-4-hydroxybenzoate. Transconjugants sucrose resistant and kanamycin sensitive were isolated and the second crossover event was confirmed by PCR using external primers and DNA sequencing.

### Construction of PIDH overexpression mutants

The pIZ3-derived vector was used to construct overexpression PIDH strains. The *icd* (PP_4011) gene was subcloned into pIZ3 (via XbaI and HindIII-HF digestion of pMM27), generating pMM39. Constructs were transformed into *P. putida* KT2440 strain. Overexpression was induced with 1 mM IPTG during growth experiments.

### Engineering of expression vectors

Expression vectors pET29a-4011, pET29a-4012, and pET29a-4012 mutants (with Ser133Ala, Asn136Ala, and Arg140Ala substitutions) were constructed using *P. putida* genomic DNA as a templates and primers listed in Table S2. PCR products were cloned into BamHI-HF and HindIII-HF digested pET29a, verified by sequencing, and transformed into *E. coli* DH10B for plasmid maintenance and BL21 (λDE3, pLysS) for protein expression induced with 1 mM IPTG.

### Overproduction and purification of recombinant isocitrate dehydrogenase

*E. coli* BL21 (λDE3, pLysS) strains harbouring pET29a constructs were cultured in fresh LB with kanamycin to an OD 600 nm of 0.6, followed by induction with 1 mM IPTG for 2 h at 37 °C. Cells were harvested, washed twice with 0.85% saline solution and pellets were stored at -80 °C until further use.

Pellets were resuspended in buffer A (50 mM NaH_2_PO_4_, 50 mM Na_2_HPO_4_, 300 mM NaCl, 20 mM imidazole at pH 7.4 and 1 tablet of Roche cOmpleteTM, EDTA free, protease inhibitor cocktail per 50 mL culture) and lysed using a French press (three passages at 1000 psi) [41]. Soluble fractions were obtained by a 30 min centrifugation at 10.000× *g* at 4 °C to remove cellular debris and were purified in a small-scale Ni-NTA spin columns (Qiagen; Catalog Number 31314), following the manufactures recommendations. Isocitrate dehydrogenase constructs were eluted at 260 mM imidazole. Imidazole was removed via dialysis using Amicon Ultra filters (0.5 mL, 10 KDa cutoff, Sigma-Aldrich, Catalog Number: Z740171) following the manufacturer's instructions. Protein concentration was measured via Bradford assay using bovine serum albumin (BSA) as a standard [42]. For analysis of the protein purity, polypeptides were separated by SDS-PAGE containing 12.5% (w/v) acrylamide, visualized by staining with Coomassie Brilliant Blue R250 and photographed.

### Isocitrate dehydrogenase enzymatic activity

Isocitrate dehydrogenase activity was measured at 37 °C to allow for direct kinetic comparisons with standardized PIDH literature values (e.g. from *Pseudomonas aeruginosa*), by monitoring NAD(P)H formation spectrophotometrically at 340 nm. Standard assays for the monomeric IDH included 25 mM Tris–HCl (pH 7.3), 5 mM magnesium chloride hexahydrate, 0.2 mM NADP^+^, 0.2 mM DL-isocitric acid trisodium salt hydrate, 10 mM sodium succinate dibasic hexahydrate (to inhibit interfering ICL activity), and 2.5 µg of purified, dialyzed enzyme. Km values for isocitrate and NADP^+^ were determined by varying their concentrations (0–0.6 mM), while keeping the other constant (0.2 mM). Because the dimeric ICD exhibited severe instability and loss of activity post-dialysis, its kinetic parameters were exclusively measured from non-dialyzed enzyme preparations directly in the phosphate elution buffer (50 mM phosphate, 300 mM NaCl, 260 mM imidazole, pH 7.4), adjusting all other cofactors to match the standard assay. To test for potential NAD⁺-dependent activity for both isoforms, identical assay conditions were utilized in triplicate, substituting 0.2 mM NADP⁺ with 0.2 mM NAD⁺. The approximate spectrophotometric limit of detection for these assays was 0.01 U/mg.

For the calculations, we considered the extinction coefficient of NADPH at 340 nm, 6.22 mM^−1^ cm^−1^, which can be used to directly quantify the product formation. Enzyme activity is expressed as U/mg, where a Unit is defined as a μmol of NAD(P)H formed per minute per milligram of protein. Graphpad prism version 6 was used for the data analysis.

### RNA extraction and qRT-PCR experiments

RNA was extracted from mid-exponential phase cultures grown in M63 medium with 10 mM fructose or 10 mM glucose, using the High Pure RNA Isolation kit (Roche) following the manufacturer’s instructions [35, 37]. RNA integrity was verified by agarose gel electrophoresis, quantified with a NanoDrop 2000 Spectrophotometer (Thermo Scientific, Massachusetts, USA), and treated with DNase (Ambion) following the manufacturer´s instructions. cDNA synthesis was performed using the Transcriptor First Strand cDNA Synthesis kit (Roche) following the manufacture´s recommendations. Briefly, cDNA was synthesized from 1 μg of purified RNA using random hexamer-primed reactions. qRT-PCR was conducted with primers listed in Table S2. Absolute quantification was used to calculate expression levels as concentration of cDNA (nM), with three technical and two independent biological replicates per condition. Absolute quantification was achieved by generating per-amplicon standard curves using serial dilutions of *P. putida* genomic DNA. Sample Cp values were interpolated against these standard curves to calculate cDNA concentration (nM), intrinsically accounting for variations in primer amplification efficiencies.

### Bioinformatic analysis

The three-dimensional structure of monomeric IDH protein was predicted using the Swiss Model program [43], with the crystal structure of monomeric PIDH from *P. aeruginosa* (PDB 6G3U) serving as the template [11]. Docking analysis to determine the positioning of the isocitrate molecule within the IDH protein was performed using AutoDock Vina via the HotSpot Wizard server [44]. The structures of the dimeric ICD, and the ICD-AceK complex were predicted using AlphaFold3 software [45]. The figures resulting from the bioinformatic analyses were created using the PyMOL software.

Additionally, the interacting proteins for both were identified using the STRING online tool (https://string-db.org/). The final interaction network was obtained by setting ICD and IDH coding genes (PP_4011 and PP_4012, respectively) as the query genes and the *P. putida* database as the protein database for inferring interactions, using a restrictive interactive score of 0.95.

### Mapping IDH architectures over a diverse bacterial sample

A representative dataset from ProGenomes4, containing more than 3200 different bacterial and archaeal genomes, was used as the primary source of assemblies, taxonomy, and ecological metadata [46]. Protein sequences were formatted into genome-aware multi-FASTA files and partitioned into ~ 50,000-sequence chunks using SeqKit [47]. Sequences exceeding 100 kb were segmented into overlapping fragments via a custom Python script to ensure compatibility with HMMER [48].

Monomeric and dimeric IDH profile HMMs were constructed from manually curated UniProt sequences supported by structural and functional annotations. Redundant or incomplete entries were removed, and multiple sequence alignments were generated and manually validated for conserved residues and motifs. These were used to build HMMs with HMMER [48].

Proteins were screened against both profiles (E-value ≤ 1 × 10⁻^5^), and classified as monomeric, dimeric, or other based on competitive bit scores. Ambiguous cases (difference < 5 bits) were labelled “other”. Genome-level architecture composition was calculated as proportions, and genomes were categorized by predominant architecture.

Taxonomic annotations were mapped automatically, and habitat data were retrieved from BacDive [49], assigning genomes to dominant habitat classes or labelling them ambiguous when no clear habitat was overrepresented. The entire workflow was implemented as a scalable Nextflow DSL2 pipeline with chunk-level and genome-level parallelization, producing integrated outputs containing protein-, genome-, taxonomy-, and architecture-level information.

### Analysis of mapped architectures using a tree-based visualization

To reduce false positives while retaining divergent homologs, hits were filtered using motif-length-normalized HMMER bit scores. Thresholds were selected empirically from the multimodal score distributions observed for each motif family, following the principle of model-specific discrimination thresholds used in curated HMM databases such as Pfam [50, 51]. A normalized cutoff of 0.7 bits per aligned position was selected as a conservative boundary separating low-scoring/background matches from high-confidence motif-containing proteins (Fig. S2).

Filtered proteins were collapsed at the taxonomic class level, and representative taxa were used to construct a phylogenetic tree based on NCBI taxonomy using the iTOL server [52]. The resulting tree was annotated for visualization with architecture composition, habitat distribution, and taxonomic representation, alongside an abundance metric reflecting class-level sampling depth (Fig. 1).

### Comparative transcriptomic analysis of ICD and IDH

All comparative transcriptomic analysis were performed using *putidaPRECISE321 dataset* from *iModulonDB* [53] as source data due to its wide range of conditions. Correlations were determined with the implementation of the Pearson correlation coefficient of *scipy* (https://docs.scipy.org/doc/scipy/reference/generated/scipy.stats.pearsonr.html) directly applied over the entire raw dataset. Also, the gene clustering according to the gene expression patterns was performed using all conditions of the dataset. For this, we used the “clustergram” component of *plotly Dash Bio* package (https://dash.plotly.com/dash-bio/clustergram) with its default setting, applying Euclidean distances for determining expression pattern similarity. The specific code implementation of this analysis can be found in the notebook present within the *GitHub ICDHyr_context* repository created for this publication (https://github.com/SBGlab/ICDHyr_context).

### Contextualized GEM solution space evaluation

For evaluating the flux solution space of ICDHyr, CS, and ICL reactions in a glycolytic and gluconeogenic metabolic state, the *iJN1462* GEM was contextualized with transcriptomic data of a study present in putidaPRECISE321dataset comparing expression profiles in glucose and myristic acid. The contextualization was performed using an in-house version of the GIMME method. The resulting function, in the first place, maps gene expression to reactions and calculates a threshold based on the quantile of expression values. Secondly, it identifies essential reactions to exclude them for the removal during the contextualization. Finally, it iterates through all GEM reactions, keeping those above the selected percentile threshold and knocking out those below it unless essential. If upon a knockout the GEM becomes infeasible, it reverts such modification. For GEM contextualization, we applied a 10th percentile expression thresh-old, meaning that genes with expression values within the lowest 10% of the distribution were removed during the contextualization procedure. The complete implementation is available in the GitHub repository, specifically in the notebook “Expression and flux analysis.ipynb”.

After GEM contextualization, flux sampling was performed over the 2 resulting models to assess all possible flux values for all reactions of interest. This was performed by using the *OptGPSampler* method available in the *cobrapy* python package. To obtain statistically relevant an unbiased results 10,000 flux values were sampled for each reaction, using a *thinning* parameter of 1000. Gene essentiality was evaluated through single-reaction knockout simulations performed in the non-contextualized reference model.

## Supplementary Information

Supplementary Material 1.

## Data Availability

Data will be made available upon request. Code and correlation analysis can be found at https://github.com/SBGlab/ICDHyr_context.

## References

[1] Kitano H. Biological robustness. Nat Rev Genet. 2004;5:826–37. 10.1038/nrg1471. 15520792

[2] Nogales J, Gudmundsson S, Duque E, Ramos L, Bernhard O. Expanding the computable reactome in *Pseudomonas putida* reveals metabolic cycles providing robustness. 2017. 10.1101/139121.

[3] Nogales J, Mueller J, Gudmundsson S, Canalejo FJ, Duque E, Monk J, et al. High‐quality genome‐scale metabolic modelling of *Pseudomonas putida* highlights its broad metabolic capabilities. Environ Microbiol. 2020;22:255–69. 10.1111/1462-2920.14843. PMC7078882 31657101

[4] Dean AM, Golding GB. Protein engineering reveals ancient adaptive replacements in isocitrate dehydrogenase. Proc Natl Acad Sci USA. 1997;94:3104–9. 10.1073/pnas.94.7.3104. PMC20329 9096353

[5] Zhu G, Golding GB, Dean AM. The selective cause of an ancient adaptation. Science. 2005;307:1279–82. 10.1126/science.1106974. 15653464

[6] Sivaraman J, Li Y, Banks J, Cane DE, Matte A, Cygler M. Crystal structure of *Escherichia coli* PdxA, an enzyme involved in the pyridoxal phosphate biosynthesis pathway. J Biol Chem. 2003;278:43682–90. 10.1074/jbc.M306344200. 12896974

[7] Spaans SK, Weusthuis RA, van der Oost J, Kengen SWM. NADPH-generating systems in bacteria and archaea. Front Microbiol. 2015;2:9. 10.3389/fmicb.2015.00742. PMC4518329 26284036

[8] Gálvez S, Gadal P. On the function of the NADP-dependent isocitrate dehydrogenase isoenzymes in living organisms. Plant Sci. 1995;105:1–14. 10.1016/0168-9452(94)04041-E.

[9] Wang P, Lv C, Zhu G. Novel type II and monomeric NAD+ specific isocitrate dehydrogenases: phylogenetic affinity, enzymatic characterization, and evolutionary implication. Sci Rep. 2015;5:1–11. 10.1038/srep09150. PMC4360740 25775177

[10] Yasutake Y, Watanabe S, Yao M, Takada Y, Fukunaga N, Tanaka I. Structure of the monomeric isocitrate dehydrogenase. Structure. 2002;10:1637–48. 10.1016/S0969-2126(02)00904-8. 12467571

[11] Crousilles A, Dolan SK, Brear P, Chirgadze DY, Welch M. Gluconeogenic precursor availability regulates flux through the glyoxylate shunt in *Pseudomonas aeruginosa*. J Biol Chem. 2018;37:14260–9. 10.1074/jbc.RA118.004514. PMC6139552 30030382

[12] Ochiai T, Fukunaga N, Sasaki S. Purification and some properties of two NADP^+^-specific isocitrate dehydrogenases from an obligately psychrophilic marine bacterium, *Vibrio* sp., strain ABE-1. J Biochem. 1979;86:377–84. 10.1093/oxfordjournals.jbchem.a132536. 39069

[13] Maki S, Yoneta M, Takada Y. Two isocitrate dehydrogenases from a psychrophilic bacterium, *Colwellia psychrerythraea*. Extremophiles. 2006;10:237–49. 10.1007/s00792-005-0493-9. 16418792

[14] Matsuo S, Shirai H, Takada Y. Isocitrate dehydrogenase isozymes from a psychrotrophic bacterium, *Pseudomonas psychrophila*. Arch Microbiol. 2010;192:639–50. 10.1007/s00203-010-0595-3. 20549192

[15] Ishii A, Suzuki M, Sahara T, Takada Y, Sasaki S, Fukunaga N. Genes encoding two isocitrate dehydrogenase isozymes of a psychrophilic bacterium, Vibrio genes encoding two isocitrate dehydrogenase isozymes of a psychrophilic bacterium, Vibrio sp. strain ABE-1. J Bacteriol. 1993;175:6873–80. 10.1128/jb.175.21.6873-6880.1993 PMC206812 8226630

[16] Suzuki M, Sahara T, Tsuruha JI, Takada Y, Fukunaga N. Differential expression in *Escherichia coli* of the *Vibrio* sp. strain ABE-1 *icdI* and *icdII* genes encoding structurally different isocitrate dehydrogenase isozymes. J Bacteriol. 1995;177:2138–42. 10.1128/jb.177.8.2138-2142.1995. PMC176858 7536733

[17] Nikel PI, Chavarría M, Danchin A, de Lorenzo V. From dirt to industrial applications: *Pseudomonas putida* as a Synthetic Biology chassis for hosting harsh biochemical reactions. Curr Opin Chem Biol. 2016. 10.1016/j.cbpa.2016.05.011. 27239751

[18] Roy SO, Packard TT. NADP-isocitrate dehydrogenase from *Pseudomonas nautica*: kinetic constant determination and carbon limitation effects on the pool of intracellular substrates. APPL Env Microbiol. 1998;64:7. 10.1128/AEM.64.12.4958-4964.1998. PMC90949 9835589

[19] Nelson KE, Weinel C, Paulsen IT, Dodson RJ, Hilbert H, Martins dos Santos VAP, et al. Complete genome sequence and comparative analysis of the metabolically versatile *Pseudomonas putida* KT2440. Environ Microbiol. 2002. 10.1046/j.1462-2920.2002.00366.x. 12534463

[20] Belda E, van Heck RGA, José Lopez-Sanchez M, Cruveiller S, Barbe V, Fraser C, et al. The revisited genome of *Pseudomonas putida* KT2440 enlightens its value as a robust metabolic chassis. Environ Microbiol. 2016. 10.1111/1462-2920.13230. 26913973

[21] Murima P, Zimmermann M, Chopra T, Pojer F, Fonti G, Dal Peraro M, et al. A rheostat mechanism governs the bifurcation of carbon flux in mycobacteria. Nat Commun. 2016. 10.1038/ncomms12527. PMC4999502 27555519

[22] Jacobs MA, Alwood A, Thaipisuttikul I, Spencer D, Haugen E, Ernst S, et al. Comprehensive transposon mutant library of *Pseudomonas aeruginosa*. Proc Natl Acad Sci USA. 2003;100:14339–44. 10.1073/pnas.2036282100. PMC283593 14617778

[23] Lewenza S. Construction of a mini-Tn5-luxCDABE mutant library in *Pseudomonas aeruginosa* PAO1: a tool for identifying differentially regulated genes. Genome Res. 2005;15:583–9. 10.1101/gr.3513905. PMC1074373 15805499

[24] Molina-Henares MA, de la Torre J, García-Salamanca A, Molina-Henares AJ, Herrera MC, Ramos JL, et al. Identification of conditionally essential genes for growth of *Pseudomonas putida* KT2440 on minimal medium through the screening of a genome-wide mutant library. Environ Microbiol. 2010. 10.1111/j.1462-2920.2010.02166.x. 20158506

[25] Martínez-García E, Nikel PI, Aparicio T, de Lorenzo V. Pseudomonas 2.0: genetic upgrading of *P. putida* KT2440 as an enhanced host for heterologous gene expression. Microb Cell Fact. 2014. 10.1186/s12934-014-0159-3. PMC4230525 25384394

[26] Yasutake Y, Watanabe S, Yao M, Takada Y, Fukunaga N, Tanaka I. Crystal Structure of the Monomeric Isocitrate Dehydrogenase in the Presence of NADP^+^: insight into the cofactor recognition, catalysis and evolution. J Biol Chem. 2003;278:36897–904. 10.1074/jbc.M304091200. 12855708

[27] Chen R, Yang H. A highly specific monomeric isocitrate dehydrogenase from *Corynebacterium glutamicum*. Arch Biochem Biophys. 2000. 10.1006/abbi.2000.2082. 11185559

[28] Kobayashi M, Takada Y. Effects of the combined substitutions of amino acid residues on thermal properties of cold-adapted monomeric isocitrate dehydrogenases from psychrophilic bacteria. Extremophiles. 2014;18:755–62. 10.1007/s00792-014-0656-7. 24913900

[29] Nagai S, Takada Y. Analysis of amino acid residues involved in the thermal properties of isocitrate dehydrogenases from a psychrophilic bacterium, *Colwellia maris*, and a psychrotrophic bacterium, *Pseudomonas psychrophila*. J Biosci Bioeng. 2019. 10.1016/j.jbiosc.2019.09.014. 31619337

[30] Watanabe S, Yasutake Y, Tanaka I, Takada Y. Elucidation of stability determinants of cold-adapted monomeric isocitrate dehydrogenase from a psychrophilic bacterium, *Colwellia maris*, by construction of chimeric enzymes. Microbiology. 2005;151:1083–94. 10.1099/mic.0.27667-0. 15817777

[31] Jordá T, Puig S. Regulation of ergosterol biosynthesis in *Saccharomyces cerevisiae*. Genes. 2020;11:795. 10.3390/genes11070795. PMC7397035 32679672

[32] Li J, Wang H-T, Wang W-T, Zhang X-R, Suo F, Ren J-Y, et al. Systematic analysis reveals the prevalence and principles of bypassable gene essentiality. Nat Commun. 2019;10:1002. 10.1038/s41467-019-08928-1. PMC6397241 30824696

[33] Sambrook J, Fritsch EF, Maniatis T. Molecular cloning, a laboratory manual. Second Edition. New York: Cold Spring Harbor Laboratory Press; 1989.

[34] Manoli M-T, Hernández-Herreros N, Rivero-Buceta V, Rodríguez A, Prieto MA. Protocol for obtaining polyhydroxyalkanoates from microbial cultures: production, quantification, and analytical assays. STAR Protoc. 2025;6:104261. 10.1016/j.xpro.2025.104261. PMC12720318 41351842

[35] Manoli M-T, Gargantilla-Becerra Á, Del Cerro Sánchez C, Rivero-Buceta V, Prieto MA, Nogales J. A model-driven approach to upcycling recalcitrant feedstocks in *Pseudomonas putida* by decoupling PHA production from nutrient limitation. Cell Rep. 2024;43:113979. 10.1016/j.celrep.2024.113979. 38517887

[36] Schäfer A, Tauch A, Jäger W, Kalinowski J, Thierbach G, Pühler A. Small mobilizable multi-purpose cloning vectors derived from the *Escherichia coli* plasmids pK18 and pK19: selection of defined deletions in the chromosome of *Corynebacterium glutamicum*. Gene. 1994. 10.1016/0378-1119(94)90324-7. 8045426

[37] Manoli M-T, Nogales J, Prieto A. Synthetic control of metabolic states in *Pseudomonas putida* by tuning polyhydroxyalkanoate cycle. mBio. 2022;13:e01794-21. 10.1128/mbio.01794-21. PMC8764540 35038900

[38] Manoli M-T, Blanco FG, Rivero-Buceta V, Kniewel R, Alarcon SH, Salgado S, et al. Heterologous constitutive production of short-chain-length polyhydroxyalkanoates in *Pseudomonas putida* KT2440: the involvement of IbpA inclusion body protein. Front Bioeng Biotechnol. 2023;11:1275036. 10.3389/fbioe.2023.1275036. PMC10646324 38026847

[39] Herrero M, De Lorenzo V, Timmis KN. Transposon vectors containing non-antibiotic resistance selection markers for cloning and stable chromosomal insertion of foreign genes in gram-negative bacteria. J Bacteriol. 1990. 10.1128/jb.172.11.6557-6567.1990. PMC526845 2172216

[40] Zobel S, Benedetti I, Eisenbach L, De Lorenzo V, Wierckx N, Blank LM. Tn7-based device for calibrated heterologous gene expression in *Pseudomonas putida*. ACS Synth Biol. 2015;4:1341–51. 10.1021/acssynbio.5b00058. 26133359

[41] Manoli M-T, Llamas P, Blanco FG, Hernández-Arriaga AM, Torices MI, Prieto MA. Exploring phasin-polyhydroxyalkanoate interactions through in vivo and in vitro binding assays. Int J Biol Macromol. 2026. 10.1016/j.ijbiomac.2026.150332. 41548774

[42] Bradford MM. A rapid and sensitive method for the quantitation of microgram quantities of protein utilizing the principle of protein-dye binding. Anal Biochem. 1976;72:248–54. 10.1016/0003-2697(76)90527-3. 942051

[43] Schwede T. SWISS-MODEL: an automated protein homology-modeling server. Nucleic Acids Res. 2003;31:3381–5. 10.1093/nar/gkg520. PMC168927 12824332

[44] Sumbalova L, Stourac J, Martinek T, Bednar D, Damborsky J. HotSpot Wizard 3.0: web server for automated design of mutations and smart libraries based on sequence input information. Nucleic Acids Res. 2018;46:W356–62. 10.1093/nar/gky417. PMC6030891 29796670

[45] Abramson J, Adler J, Dunger J, Evans R, Green T, Pritzel A, et al. Addendum: accurate structure prediction of biomolecular interactions with AlphaFold 3. Nature. 2024;636:E4–E4. 10.1038/s41586-024-08416-7. PMC11634763 39604737

[46] Fullam A, Letunic I, Maistrenko OM, Castro AA, Coelho LP, Grekova A, et al. ProGenomes4: providing 2 million accurately and consistently annotated high-quality prokaryotic genomes. Nucleic Acids Res. 2026;54:D852–7. 10.1093/nar/gkaf1208. PMC12807698 41261732

[47] Shen W, Sipos B, Zhao L. SeqKit2: a Swiss army knife for sequence and alignment processing. iMeta. 2024;3:e191. 10.1002/imt2.191. PMC11183193 38898985

[48] Rajković A, Beracochea M, Rogers AB, Eddy SR, Carter NP, Finn RD. HMMER web server: 2026 update. Nucleic Acids Res. 2026. 10.1093/nar/gkag373. PMC13355042 42037125

[49] Schober I, Koblitz J, Sardà Carbasse J, Ebeling C, Schmidt ML, Podstawka A, et al. Bac *dive* in 2025: the core database for prokaryotic strain data. Nucleic Acids Res. 2025;53:D748–56. 10.1093/nar/gkae959. PMC11701647 39470737

[50] Mistry J, Chuguransky S, Williams L, Qureshi M, Salazar GA, Sonnhammer ELL, et al. Pfam: the protein families database in 2021. Nucleic Acids Res. 2021;49:D412–9. 10.1093/nar/gkaa913. PMC7779014 33125078

[51] Srivastava PK, Desai DK, Nandi S, Lynn AM. HMM-ModE – improved classification using profile hidden Markov models by optimising the discrimination threshold and modifying emission probabilities with negative training sequences. BMC Bioinform. 2007;8:104. 10.1186/1471-2105-8-104. PMC1852395 17389042

[52] Letunic I, Bork P. Interactive tree of life (iTOL) v6: recent updates to the phylogenetic tree display and annotation tool. Nucleic Acids Res. 2024;52:W78-82. 10.1093/nar/gkae268. PMC11223838 38613393

[53] Catoiu EA, Krishnan J, Li G, Lou XA, Rychel K, Yuan Y, et al. iModulonDB 2.0: dynamic tools to facilitate knowledge-mining and user-enabled analyses of curated transcriptomic datasets. Nucleic Acids Res. 2025;53:D99-106. 10.1093/nar/gkae1009. PMC11701608 39494532

[54] Bagdasarian M, Lurz R, Rückert B, Franklin FC, Bagdasarian MM, Frey J, et al. Specific-purpose plasmid cloning vectors. II. Broad host range, high copy number, RSF1010-derived vectors, and a host-vector system for gene cloning in *Pseudomonas*. Gene. 1981;16:237–47. 10.1016/0378-1119(81)90080-9. 6282695

[55] Boyer HW, Roulland-Dussoix D. A complementation analysis of the restriction and modification of DNA in *Escherichia coli*. J Mol Biol. 1969;41:459–72. 10.1016/0022-2836(69)90288-5. 4896022

[56] Kessler B, de Lorenzo V, Timmis KN. A general system to integrate *lacZ* fusions into the chromosomes of gram-negative eubacteria: regulation of the Pm promoter of the TOL plasmid studied with all controlling elements in monocopy. Mol Gen Genet. 1992;233:293–301. 10.1007/BF00587591. 1318499

[57] Schäfer A, Tauch A, Jäger W, Kalinowski J, Thierbach G, Pühler A. Small mobilizable multi-purpose cloning vectors derived from the *Escherichia coli* plasmids pK18 and pK19: selection of defined deletions in the chromosome of *Corynebacterium glutamicum*. Gene. 1994;145:69–143. 10.1016/0378-1119(94)90324-7. 8045426

[58] Acedos MG, De la Torre I, Santos VE, García-Ochoa F, García JL, Galán B. Modulating redox metabolism to improve isobutanol production in *Shimwellia blattae*. Biotechnol Biofuels. 2021;14:1–11. 10.1186/s13068-020-01862-1. PMC7789792 33407735

