# Supplementary material for: Functional redundancy driven by isocitrate dehydrogenase enhances metabolic robustness in Pseudomonas putida KT2440
Source: Microb Cell Fact. 2026 Jul 16;25:207. doi: 10.1186/s12934-026-03053-3 (PMC13660168; doi:10.1186/s12934-026-03053-3)
Supplement: Supplementary file 1 [file 12934_2026_3053_MOESM1_ESM.docx]

**Functional redundancy driven by isocitrate dehydrogenase enhances metabolic robustness in *Pseudomonas putida* KT2440**

Maria-Tsampika Manoli,^1,2^ Sandra Herrera Alarcon,^1,2*^ Álvaro Gargantilla-Becerra,^3^ M. Auxiliadora Prieto,^1,2#^ and Juan Nogales^2,3,4#^

1. Polymer Biotechnology Group, Department of Biotechnology, Biological Research Centre Margarita Salas (CIB-CSIC), Madrid, Spain

2. Interdisciplinary Platform for Sustainable Plastics towards a Circular Economy-Spanish National Research Council (SusPlast-CSIC), Madrid, Spain

3. Systems Biotechnology Group (SBG), Department of Systems Biology, Centro Nacional de Biotecnología (CNB-CSIC), Madrid, Spain

4. CNB DNA Biofoundry (CNBio), CSIC, National Centre for Biotechnology (CNB-CSIC), Madrid, Spain

#Address correspondence to Juan Nogales ([jnogales@cnb.csic.es](mailto:jnogales@cnb.csic.es)) and M. Auxiliadora Prieto ([auxi@cib.csic.es](mailto:auxi@cib.csic.es)).

*Present address: CBM Severo Ochoa-CSIC, Tissue and organ homeostasis Scientific program.

**Supplementary Table S1.** Selected examples of bacteria with dimeric, monomeric, and both NADP^+^ isocitrate dehydrogenase are listed.

| Organism | Reference |
| --- | --- |
| **Dimeric** | |
| *Bacillus stearothermophilus* | [1] |
| *Bacillus subtilis* | [2,3] |
| *Bifidobacterium longum* | [4] |
| *Escherichia coli* | [5,6] |
| *Methylobacillus flagellatus* | [7] |
| *Micromonas sp* RCC299 | [8] |
| *Ostreococcus lucimarinus* CCE9901 | [8] |
| *Streptococcus mutans* | [9] |
| *Thermus thermophilus HB8* | [10] |
| *Zymomonas mobilis* | [11] |
| **Monomeric** | |
| *Acinetobacter baumannii* | [12] |
| *Acinetobacter calcoaceticus* | [13,14] |
| *Azotobacter vinelandii* | [6,15,16] |
| *Chlorobium limicola* | [17] |
| *Corynebacterium glutamicum* | [18,19] |
| *Desulfobacter vibrioformis* | [20] |
| *Psychromonas marina* | [21] |
| *Rhizobium meliloti* | [22] |
| *Streptomyces lividans* TK54 | [23] |
| *Vibrio parahaemolyticus* | [24] |
| **Monomeric and Dimeric** | |
| *Colwellia maris* (formally *Vibrio* sp ABE-1) | [6,25–29] |
| *Colwellia psychrerythraea* NRC1004 | [29,30] |
| *Colwellia psychrerythraea* 34H | [31] |
| *Cupriavidus necator* | [32] |
| *Mycobacterium bovis* | [33] |
| *Mycobacterium tuberculosis* | [33] |
| *Pseudomonas aeruginosa* PAO1 | [34] |
| *Pseudomonas nautica* | [35] |
| *Pseudomonas psychrophila* | [36] |
| *Xylella fastidiosa* | [37] |

Supplementary Table S2. Oligonucleotides used in this work. Underlined sequence means the restriction site for restriction enzymes, with bold capital letters the point mutations are shown. The following restriction enzymes were used: BamHI-HF (GGATCC), EcoRI-HF (GAATTC), HindIII-HF (AAGCTT).

| **Oligonucleotide** | **Sequence (5´-3´)** | **Utility** | **Affected genomic locus**  **/Reference** |
| --- | --- | --- | --- |
| A-For | TTTAAGCTTATTTCATCATGAGCGTCAGGTTA | Cloning PCR | PP_4011  deletion, [38] |
| A-Rev | GACAGCGGAGTAGTGCAAGCAGAAAACCGGTCGCTTCTTTTAA | Cloning PCR |  |
| B-For | GCTTGCACTACTCCGCTGTC | Cloning PCR |  |
| B-Rev | TTTGGATCCCGGGTTAACTCTCTGTGTGCTG | Cloning PCR |  |
| A-Sec | AATCCATTGGTGTGTAGTCATCG | Sequencing primer, Genome PCR verification |  |
| A-Sec-Ins | GGTATTCAACATCGTCCAGGG | Sequencing primer, Genome PCR verification |  |
| MM152 | CATAAGCTTCATGTGTTTGATCATTTCATCGC | Cloning PCR of PP_4011 to pET29a | PP_4011 |
| MM153 | CATGGATCCATGGGATACCAGAAAATCAAGGTTC | Cloning PCR of PP_4011 to pET29a |  |
| MM178 | AACTCCGAAGACATCTATGCCG | qRT-PCR_PP_4011 |  |
| MM201 | CTTGGTACCCTCCTTCGACAC | qRT-PCR_PP_4011 |  |
| MM330 | TTTGGATCCATGCCCACCCGTTCCAAGATCAT | Cloning PCR of PP_4012 to pET29a | PP_4012 |
| MM331 | TTTAAGCTTCAGGGCAGCGATGGCGCT | Cloning PCR of PP_4012 to pET29a |  |
| MM223 | G**GC**GTCGGAGTTGCCTTCG**GC**CAGCACCGGG**GC**CACGGCGG**C**GCCCTTGATGCGGTCGTAG | Cloning PCR of PP_4012-point mutations (Ser133Ala; Asn136Ala; Arg140Ala) to pET29a |  |
| MM224 | **G**CCGCCGTG**GC**CCCGGTGCTG**GC**CGAAGGCAACTCCGAC**GC**C | Cloning PCR of PP_4012-point mutations (Ser133Ala; Asn136Ala; Arg140Ala) to pET29a |  |
| MM332 | GCACCGGGTTCACGGCGGCGCCCTTGATGCGGTC | Cloning PCR of PP_4012_Ser133Ala (T>G) to pET29a |  |
| MM333 | GCCGCCGTGAACCCGGTGC | Cloning PCR of PP_4012_Ser133Ala (T>G) to pET29a |  |
| MM334 | CTTCGCGCAGCACCGGGGCCACGGCGGAGCCCTTG | Cloning PCR of PP_4012_Asn136Ala (AA>GC) to pET29a |  |
| MM335 | GCCCCGGTGCTGCGCGAAG | Cloning PCR of PP_4012_Asn136Ala (AA>GC) to pET29a |  |
| MM179 | GCAAGCACCCACACAAGATG | qRT-PCR_PP_4012 (*idh*) |  |
| MM202 | CGGTCTTTTCTTTCAGCACGG | qRT-PCR_PP_4012 (*idh*) |  |
| MM158 | CATCTACCAGGCCACCATCG | PP_4012_Sequencing primer |  |
| MM225 | GCCCCGGTGCTGGC | PP_4012_Sequencing primer |  |
| MM226 | GTCCTTGATGCGGGCGTA | PP_4012_Sequencing primer |  |
| MM395  MM396 | GTCAAACGCATCAACAACGC  CGCCTGCTTCGATCATGTTC | *aceA*, PP_4116  *aceA*, PP_4116 | PP_4116 |
| T7prom | TAATACGACTCACTATAGGG | pET29a (+) sequencing primer | - |
| TT7 | GCTAGTTATTGCTCAGCGG | pET29a (+) sequencing primer | - |

**Fig. S1. Genomic localization of isocitrate dehydrogenase genes in various bacteria.** Monomeric and dimeric are shown in blue and orange, respectively. I. Isocitrate dehydrogenase genes in psychrophilic microorganisms. II. Mesophilic microorganisms with two isocitrate dehydrogenase genes. In case of *P. putida* KT2440, ICD: PP_4011 locus, and IDH: PP_4012. III. Examples of microorganisms with a single isocitrate dehydrogenase gene.

Fig. S2. Scores of mapped IDH architectures over Progenomes4 database. Histograms showing the obtained results for the mapping of dimeric (A) and monomeric (B) IDH architectures over progenomes4 database. The normalized score threshold is contextualized for both cases using a dashed line.

**Fig. S3** Growth curves of KT2440 derived strains: KT2440 (wild type WT, green), KT2440 (pMM39) (light orange, *icd* overexpressing), and Δ*idh*/PP_4012 (pMM39) (dark blue, *idh* null, *icd* overexpressing). Growth was evaluated in: **A.** LB medium, **B.** LB with 15 mM α-ketoglutarate, **C.** M63 with 15 mM α-ketoglutarate, **D.** M63 with 10 mM glucose. These experiments correspond to one biological replicate, except for the case of Δ*idh*/PP_4012 (pMM39) were the data correspond to the mean and standard deviation values of the three mutant clones.

```
M. tuberculosis      -MSNAPKIKVSGPVVELDGDEMTRVIWKLIKDMLILPYLDIRL---D-------YYDLGI 49
C. maris             -MTNKIIIPTTGDKITFI-DGKL-----SVPNNPIIPYIEGDGIGVDVTPPMLKVVNAAV 53
C. psychrerythraea   -MTNQIIIPTTGDKITFV-DGKL-----SVPNNPIIPFIEGDGIGVDVTPPMLKVVNAAV	53
E. coli K-12         -MESKVVVPAQGKKITLQ-NGKL-----NVPENPIIPYIEGDGIGVDVTPAMLKVVDAAV	53
P. aeruginosa PAO1   MGYQKIQVPATGDKITVNADMSL-----SVPKNPIIPFIEGDGIGVDISPVMIKVVDAAV	55
P. psychrophila      MGYNKIKVPAAGEKITVNADHSL-----NVPDNPIIPYIEGDGIGVDISPVMIKVVDAAV  55
P. putida KT2440     MGYQKIKVPTDGTKITVNADHSL-----NVPDHPIIPYIEGDGIGVDVSPVMIKVVDAAV  55
  .   : . *  : .  :         : .  *:*:::      *         : .:

M. tuberculosis      EHRDATDDQVTIDAAYAIKKHGVGVKCATITPDEARVEEFNLKKMWLSPNGTIRNILGGT	 109
C. maris             AKAYGGDRKIEWLEVYAGEKATKMYDSETWLPEETL---NILQEYKVSIKGPLTTPVGGG	 110
C. psychrerythraea   AKAYAGEREIAWMEVYAGEKATKMYDSETWLPEETL---AILQEYKVSIKGPLTTPVGGG	 110
E. coli K-12         EKAYKGERKISWMEIYTGEKSTQVYGQDVWLPAETL---DLIREYRVAIKGPLTTPVGGG	 110
P. aeruginosa PAO1   EKAYKGERKIAWMEVYAGEKATQVYDQDTWLPQETL---DAVRDYVVSIKGPLTTPVGGG	 112
P. psychrophila      SKAYAGKRKISWMEVYAGEKATQVYDQDTWLPQETL---DAVKDYVVSIKGPLTTPVGGG	 112
P. putida KT2440     QKAYGGKRKIAWMEVYAGEKATQVYDQDTWLPQETL---DAVRDYVVSIKGPLTTPVGGG	 112
:    . ::     *: :*        .  * *:      ::.  :: :* : . :**

M. tuberculosis      IFREPIVIS----------------NVPR--LVPGWTKPIVIGRHAFGDQYRATNFKVDQ	 151
C. maris             MSSLNVAIRQMLDLYVCQRPVQWFTGVPSPVKRPSE-VDMVIFRENTEDIYAGIEYKAGS	 169
C. psychrerythraea   MSSLNVAIRQMLDLYVCQRPVQWFTGVPSPVKRPSE-VDMVIFRENTEDIYAGIEYKAGS	 169
E. coli K-12         IRSLNVALRQELDLYICLRPVRYYQGTPSPVKHPEL-TDMVIFRENSEDIYAGIEWKADS	 169
P. aeruginosa PAO1   IRSLNVALRQQLDLYVCQRPVRWFEGVPSPVKKPGD-VDMVIFRENSEDIYAGVEWKAGS	 171
P. psychrophila      IRSLNVALRQQLDLYVCLRPVRWFEGVPSPVKKPGD-VDMTIFRENSEDIYAGIEWKAGS	 171
P. putida KT2440     IRSLNVALRQQLDLYVCLRPVLWFQGVPSPVKKPGD-VDMVIFRENSEDIYAGIEWKAGS	 171
      :    :.:                 ..*     *     :.* *.   * * . ::*...

M. tuberculosis      PGTVTL-TFTPADGSAPIVHEMVSIPEDGGVVLGMYNFKESIRDFARASFSYGLN-AKWP	 209
C. maris             DKAKSVIKFLIEEM----GASNIRFTENCGIGIKPV-SKEGSQRLVRQAIQYAIDNNKDS	 224
C. psychrerythraea   DKAKAVIEFLTEEM----GASNIRFTDNCGIGIKPV-SKEGSQRLVRQAIQYAIDNDKDS	 224
E. coli K-12         ADAEKVIKFLREEM----GVKKIRFPEHCGIGIKPC-SEEGTKRLVRAAIEYAIANDRDS	 224
P. aeruginosa PAO1   PEAEKVIKFLTEEM----GVKKIRFTENCGIGIKPV-SQEGTKRLVRKALQYAVDNDRSS	 226
P. psychrophila      PEAIKVIKFLKEEM----GVTKIRFDQDCGIGVKPV-SKEGTQRLARKALQYVVDNDRES	 226
P. putida KT2440     PEANKVIKFLKEEM----GVTKIRFDQDCGIGVKPV-SKEGTKRLVRKALQYVVDNDRES	 226
        :  :  *   :         : : :. *: :     :*. : :.* ::.* :   :

M. tuberculosis      VYLSTKNTILKAYDGMFKDEFERVYEEEFKAQFEAAGLTY-------------EHRLIDD	 256
C. maris             VTLVHKGNIMKFTEGAFKDWGYELAIEEFGASLLHGGPWCSLKNPNTGKEIIIKDVIADA	 284
C. psychrerythraea   VTLVHKGNIMKFTEGAFKDWGYELARDEFGASLIDGGPWSTLTNPNTGNEIIIKDVIADA	 284
E. coli K-12         VTLVHKGNIMKFTEGAFKDWGYQLAREEFGGELIDGGPWLKVKNPNTGKEIVIKDVIADA	 284
P. aeruginosa PAO1   VTLVHKGNIMKFTEGAFKDWGYEVARDEFGAELLDGGPWMQFKNPKTGKNVVVKDVIADA	 286
P. psychrophila      LTIVHKGNIMKFTEGAFKEWAYEVAAEEFGATLLDGGPWMQFKNPKTGRNVIVKDAIADA	 286
P. putida KT2440     LTLVHKGNIMKFTEGAFKDWGYEVARDEFGAELLDGGPWMKFKNPKTGREVIVKDAIADA	 286
      : :  *..*:*  :* **:   .:  :** . :  .*                :. : *

M. tuberculosis      MVAACLKW-EGGYVWACKNYDGDVQSDTVAQGYGSLGLMTSVLMTADGKTVEAEAAHGTV	 315
C. maris             MLQQVLLRPAEYSVIATLNLNGDYLSDALAAQVGGIGIAPGANLGDEVA--VFEATHGTA	 342
C. psychrerythraea   MLQQILLRPAEYSVIATLNLNGDYLSDALAAQVGGIGIAPGANLGDEVA--VFEATHGTA	 342
E. coli K-12         FLQQILLRPAEYDVIACMNLNGDYISDALAAQVGGIGIAPGANIGDECA--LFEATHGTA	 342
P. aeruginosa PAO1   MLQQILLRPAEYDVIATLNLNGDYLSDALAAEVGGIGIAPGANLSDSVA--MFEATHGTA	 344
P. psychrophila      MLQQILLRPAEYDVIATLNLNGDYLSDALAAEVGGIGIAPGANLSDTIA--MFEATHGTA	 344
P. putida KT2440     MLQQILLRPAEYDVIATLNLNGDYLSDALAAEVGGIGIAPGANLSDTVA--MFEATHGTA	 344
      ::   *       * *  * :**  **::*   *.:*:  .. :         **:***.

M. tuberculosis      TRHYRQYQAGKPTSTNPIASIFAWTRGLQHRGKLDGTPEVIDFAHKLESVVIATVESGKM	 375
C. maris             PKYAGKN------KVNPGSVILSAEMMLRHMGWLEA-DLLLKGMS-------GAIQAKTV	 388
C. psychrerythraea   PKYAGKN------KVNPGSVILSAEMMLRHMGWLEAADLLLKGMS-------GAIKAKTV	 389
E. coli K-12         PKYAGQD------KVNPGSIILSAEMMLRHMGWTEAADLIVKGME-------GAINAKTV	 389
P. aeruginosa PAO1   PKYAGQD------KVNPGSLILSAEMMLRHMGWTEAADLIIKGTN-------GAIAAKTV	 391
P. psychrophila      PKYAGKD------QVNPGSLILSAEMMLRHMGWVEAADLIIKGTN-------GAISAKTV	 391
P. putida KT2440     PKYAGQD------KVNPGSVILSAEMMLRHMGWTEAADLIIKGTN-------GAIAAKTV	 391
                      ::  :       ..** : *::    *:* *  :.   ::.          .:: : .:
M. tuberculosis      TKDLAILIGPEQDWLNSEEFLDAIADNLEKELAN 409
C. maris             TYDFERLM-DDATLVSCSAFGDCIIDHM------ 415
C. psychrerythraea   TYDFERLM-DDATLVSCSAFGDCIIDHM------ 416
E. coli K-12         TYDFERLM-DGAKLLKCSEFGDAIIENM------ 416
P. aeruginosa PAO1   TYDFERLM-DGATLLSCSEFGDAMIAKM------ 418
P. psychrophila      TYDFERLM-DGATLVSSSGFGDALIAHM------ 418
P. putida KT2440     TYDFERLM-EGAKLVSSSGFGDEMIKHM------ 418
      * *:  *:      :... * * :  ::
```

**Fig. S4. Dimeric isocitrate dehydrogenase multiple alignment using ClustalW.** The amino acids that confer NADP^+^- coenzyme specificity and are responsible for metal (Mg^2+^, Mn^2+^) and substrate binding are indicated in shaded pink, light green and blue colors, respectively. Phosphorylation sites by AceK are indicated in bold letters. * (asterisk) indicates positions, which have a single, fully conserved residue; : (colon) indicates conservation between groups of strongly similar properties - scoring > 0.5 in the Gonnet PAM 250 matrix; . (period) indicates conservation between groups of weakly similar properties - scoring =< 0.5 in the Gonnet PAM 250 matrix. The database accession numbers are for *C. maris* (BAA03135), *C. psychrerythraea* 34H (AAZ24108), *P. psychrophila* (BAH80316), *P. aeruginosa* PAO1 (AAG06011), *P. putida* KT2440 (NP_746141), *M. tuberculosis* H37Rv (NP_217856), and *E. coli* K12 (BAA35958).

```
C. maris         MSTDNSKIIYTITDEAPALATYSLLPIIQAYTASSGINVETRDISLAGRILANFPKYLTK 60
34H              MSTDNSKIIYTITDEAPALATYSLLPIIQAYTASSGINVETRDISLAARILANFPKYLTK 60
A.vinelandii     --MSTPKIIYTLTDEAPALATYSLLPIIKAFTGSSGIAVETRDISLAGRLIATFPEYLTD 58
P. psychrophila  -MPNRSKIIYTFTDEAPALATYSLLPIIEAFTAPADIAVETRDISLAGRILASFPEQLGA 59
P. putida KT2440 -MPTRSKIIYTFTDEAPALATYSLLPIIEAFTASADIAVETRDISLAGRILAAFPEQLGA 59
P. aeruginosa    -MSIRSKITYTFTDEAPALATYSLLPIVKAFAASAGIDVETSDISLAGRILANFADRLEA 59
34H_2            ---MSSKIIYTYTDEAPLLATCSLLPIIKTFAASADIDIELKDISLASRVLSAFSDILPA 57
M. tuberculosis  MSAEQPTIIYTLTDEAPLLATYAFLPIVRAFAEPAGIKIEASDISVAARILAEFPDYLTE 60
                       .* ** ***** *** ::***:.:::  :.* :*  ***:*.*::: * . *

C. maris         EQRIDDALAELGELAQTPEANIIKLPNISASIPQLEAVIKELQAKGYDLPHYPAEPQNEA 120
34H              EQRVDDALSELGKLAKTPEANIIKLPNISASIPQLELAIKELQAKGYDLPHYPAEPQNEA 120
A.vinelandii     TQKISDDLAELGKLATTPDANIIKLPNISASVPQLKAAIKELQQQGYKLPDYPEEPKTDT 118
P. psychrophila  K-AVADHLAELGALAVTPEANIIKLPNISASVPQLQAAIKELQAQGFDIPDYPETVTTDA 118
P. putida KT2440 EKQVGDHLAELGQLATTPEANIIKLPNISASVPQLKAAIKELQGKGFNIPDYADEPATAE 119
P. aeruginosa    DQRIEDDLARLAVLATSPDANIIKLPNISASVPQLKGAIAELQGLGYKVPDFPEDPQTDE 119
34H_2            DQQVPDTLAELGALTHHPDTNIIKLPNISASIPQLKDVISELQAQGYAIPDYPESPKTDE 117
M. tuberculosis  EQRVPDNLAELGRLTQLPDTNIIKLPNISASVPQLVAAIKELQDKGYAVPDYPADPKTDQ 120
                    : * *:.*. *:  *::***********:***  .* ***  *: :*.:     .

C. maris         EESIKLTYAKILGSAVNPVLREGNSDRRAPASVKQYARNNPHSMGAWSKESKSHVAHMAS 180
34H              EESIKLTYAKVLGSAVNPVLREGNSDRRAPGSVKQYARNNPHSMGAWSPESKSHVAHMES 180
A.vinelandii     EKDVKARYDKIKGSAVNPVLREGNSDRRAPLSVKNYARKHPHKMGAWSADSKSHVAHMDN 178
P. psychrophila  DKEVKARYSKVMGSAVNPVLREGNSDRRAPLSVKNYARKHPHKMGAWAADSKSHVAHMSE 178
P. putida KT2440 EKESRARYDRIKGSAVNPVLREGNSDRRAPLSVKNYARKHPHKMGAWAADSKSHVAHMTQ 179
P. aeruginosa    EKEVRARYAKILGSAVNPVLREGNSDRRAPAAVKAYARKHPHSMGKWSMASRSHADYMRG 179
34H_2            DRSNEAKYSKTLGSAVNPVLREGNSDRRAPPSVKAFARKNPHSMGKWSQASRTHVSHMHS 177
M. tuberculosis  EKAIKERYARCLGSAVNPVLRQGNSDRRAPKAVKEYARKHPHSMGEWSMASRTHVAHMRH 180
                 :.  .  * :  *********:******** :** :**::**.** *:  *::*. :*


C. maris         GDFYGSEKSVTIDGATSVNIEFVAKNG-DVTLLKSKLPLLDKEIIDASVMSKSALVEFFE 239
34H              GDFYGSEKSVTIDGATSINIEFVAQNG-DVTLLKSKLPLLDKEIIDASVMSKSALIEFYE 239
A.vinelandii     GDFYGSEKAALIGAPGSVKIELIAKDG-SSTVLKAKTSVQAGEIIDSSVMSKNALRNFIA 237
P. psychrophila  GDFYASEKAALIEAPGSVKIELIAQDG-TTTVLKEKTAVQAGEIIDCSVMSKKALRQFVA 237
P. putida KT2440 GDFYGSEKAALIEADDSLRIELVGKDG-STTVLKEKTAVKAAEVIDCATMSRKALKAFIA 238
P. aeruginosa    GDFFSSEQSITMAKAGDVRIEFVGKDG-K-VEVKKQLSLQEGEVLDSMFMSCGKLRDFFE 237
34H_2            GDFYASEKCMTMDKACDVKIEFVAKDDGAVTVLKEKTSLQAGEIIDSMFMSKKILCEFLE 237
M. tuberculosis  GDFYAGEKSMTLDRARNVRMELLAKS-GKTIVLKPEVPLDDGDVIDSMFMSKKALCDFYE 239
                 ***:..*:.  :    .:.:*::.:.      :* :  :   :::*.  **   *  *

C. maris         TEINKAKEEDVLLSLHLKATMMKVSDPVMFGHAVRVFYKDVFAKHAATFEQLGVDADNGI 299
34H              TEINKAKEEDVLLSLHLKATMMKVSDPIMFGHAVKVFYKDVFTKHEATFDQLGVDADNGI 299
A.vinelandii     AEIEDAKKQGVLLSVHLKATMMKVSDPIMFGQIVSEFYKDALTKHAEVLKQIGFDVNNGI 297
P. psychrophila  AEIEDAKKQGVLFSVHLKATMMKVSDPIMFGQIVAEFYKDALEKHADILKEIGFNLNNGI 297
P. putida KT2440 EQIADAKAAGVLLSVHLKATMMKVSDPIMFGVIVEEFYNDVLTKHAAALAEVGFNANNGI 298
P. aeruginosa    KTLQDCKETGVMWSLHVKATMMKISHPIVFGHAVSVYYKDVFDKWGQLFEELGVNPNNGI 297
34H_2            QEMEDARETGVMFSFHVKATMMKVSHPIVFGHAVKVYYKDLFAKHAETFEKLGVNANDGV 297
M. tuberculosis  EQMQDAFETGVMFSLHVKATMMKVSHPIVFGHAVRIFYKDAFAKHQELFDDLGVNVNNGL 299
                   : ..   .*: *.*:******:*.*::**  *  :*:* : *    : .:*.: ::*:

C. maris         GDVYAKIARLPAAQKEEIEADLQAVYATRPEMAMVDSDKGITNLHVPSDVIIDASMPAAL 359
34H              GDVYAKIARLPAAQKAEIEADLQAVYATRPEMAMVDSDKGITNLHVPSDVIIDASMPAAL 359
A.vinelandii     GDLYARIKTLPEAKQKEIEADIQAVYAQRPQLAMVNSDKGITNLHVPSDVIVDASMPAMI 357
P. psychrophila  GDLYARIKALPAEKQAEIEADIQAVYANRPALAMVNSDKGITNLHVPSDVIVDASMPAMI 357
P. putida KT2440 GDLYARIKDLPADKQAEIEADIQALYAERPALAMVNSDKGITNLHVPSDVIVDASMPAMI 358
P. aeruginosa    SSVYDKIKSLPASQQEEILHDIHEVYSHRPEMAMVDSVKGITNLHIPSDVIVDASMPAMI 357
34H_2            SSIYEKVKQLPESWREEILADLHACYEKRPELAMVDSAKGISNLHTPNEVIVDASMPAMI 357
M. tuberculosis  SDLYSKIESLPASQRDEIIEDLHRCHEHRPELAMVDSARGISNFHSPSDVIVDASMPAMI 359
                 ..:* ::  **   : **  *::  :  ** :***:* :**:*:* *.:**:****** :

C. maris         RASGMMWGPDGKQKDTKFMIPDRNYAGVFSAVVDFCRENGAFNPATMGTVPNVGLMAQKA 419
34H              RASGMMWAPDGSQKDTKFMIPDRNYAGVFSAVVDFCRENGAFNPTTMGTVPNVGLMAQKA 419
A.vinelandii     RDSGKMWGPDGKLHDTKAVIPDRCYAGVYQVVIEDCKQHGAFDPTTMGSVPNVGLMAQKA 417
P. psychrophila  RDSGKMWNTEGQLQDTKAVIPDRCYATIYQATIEDCQKHGAFDPTTMGSVPNVGLMAQKA 417
P. putida KT2440 RDSGKMWNAAGELQDAKAVIPDRCYAGIYQATIEDCKVNGAFDPTTMGSVPNVGLMAQKA 418
P. aeruginosa    RNSGQMWGKDGKQKDTKAVMPESTYARIYQEMINFCKTNGAFDPTTMGSVPNVGLMAQKA 417
34H_2            RAGGKMWGPDGKLKDTKAVMPESTFARIYQEIINFCKTHGAFDPTTMGTVPNVGLMAQKA 417
M. tuberculosis  RAGGKMYGADGKLKDTKAVNPESTFSRIYQEIINFCKTNGQFDPTTMGTVPNVGLMAQQA 419
                 * .* *:   *. :*:* : *:  :: ::.  :: *: :* *:*:***:*********:*
```

```
C. maris         EEYGSHDKTFTMKAAGTVRVVN-SQGERLIEQEVAQGDIYRMCQVKDAPIQDWVKLAVTR 478
34H              EEYGSHDKTFTMSATGVVRVVN-DNGDILIEQNVAQGDIFRMCQVKDAPIQDWVKLAVTR 478
A.vinelandii     EEYGSHDKTFQIPADGVVRVTD-ESGKLLLEQSVEAGDIWRMCQAKDAPIQDWVKLAVNR 476
P. psychrophila  EEYGSHDKTFQIKTPGVVRVTD-SNGNVLMEQNVEEGDIWRMCQAKDAPIRDWVKLAVNR 476
P. putida KT2440 EEYGSHDKTFQIKADGVVRVVD-GKGNVVLEQNVEAGDIFRMCQTKDAPIQDWVKLAVNR 477
P. aeruginosa    EEYGSHDKTFEMTADGTMRVVL-ADGSVLMQHKVETGDIWRACQTKDAPIRDWVKLAVTR 476
34H_2            EEYGSHDKTFEVQGDGCIRIVDD-EGKTLLTMDVEEGDIWRMCQVKDAPIQDWVKLAVAR 476
M. tuberculosis  EEYGSHDKTFEIPEDGVANIVDVATGEVLLTENVEAGDIWRMCIVKDAPIRDWVKLAVTR 479
                 ********** :   *  .:.    *. ::  .*  ***:* * .*****:******* *

C. maris         ARATGTPTVFWLDENRGHDEQMIKKVNTYLADHDTTGLDIQILEPVKACEFTLARVAKGE 538
34H              AKATGTPTVFWLDENRGHDAEMIKKVNTYLADHDTNGLDIQILEPVKACEYTLARVAKGE 538
A.vinelandii     ARATNTPAVFWLDPARAHDAQVIAKVERYLKDYDTSGLDIRILSPVEATRFSLARIREGK 536
P. psychrophila  ARASNTPAVFWLDPKRSHDAEMIKKVETYLKDHDTSGLDIRIMAPVDAMKFTLERTRAGL 536
P. putida KT2440 ARLSNTPAVFWLDPARAHDGVMIEKVQKYLKDHDTTGLDIRILAPVDAIKFSLARIREGK 537
P. aeruginosa    ARQSDTPAIFWLDPERAHDRELRKKVELYLKDHDLTGLDISIMGYNEAIRVSMERLIRGK 536
34H_2            AKLSDTPAVFWLDEYRPHEAELIKKVKLYLKDHDLTGLDIQIMSQTRAMRYTLERILRGG 536
M. tuberculosis  ARISGMPVLFWLDPYRPHENELIKKVKTYLKDHDTEGLDIQIMSQVRSMRYTCERLVRGL 539
                 *: :. *.:****  * *:  :  **: ** *:*  **** *:    : . :  *   *

C. maris         DAISVTGNVLRDYLTDLFPILELGTSAKMLSIVPLMNGGGLFETGAGGSAPKHVQQFEKE 598
34H              DAISVTGNVLRDYLTDLFPILELGTSAKMLSIVPLMNGGGLFETGAGGSAPKHVQQFEKE 598
A.vinelandii     DTISVTGNVLRDYLTDLFPIMELGTSAKMLSIVPLMSGGGLFETGAGGSAPKHVQQFLEE 596
P. psychrophila  DTISVTGNVLRDYLTDLFPIMELGTSAKMLSIVPLMNGGGLFETGAGGSAPKHVQQLLEE 596
P. putida KT2440 DTISVTGNVLRDYLTDLFPIMELGTSAKMLSIVPLMNGGGLFETGAGGSAPKHVQQLVEE 597
P. aeruginosa    DTISVTGNVLRDYLTDLFPIMELGTSAKMLSIVPLMAGGGMYETGAGGSAPKHVQQLVEE 596
34H_2            DTISVTGNILRDYLTDLFPILELGTSAKMLSIVPLMAGGGLFETGAGGSAPKHVKQLIEE 596
M. tuberculosis  DTIAATGNILRDYLTDLFPILELGTSAKMLSVVPLMAGGGMYETGAGGSAPKHVKQLVEE 599
                 *:*:.***:***********:**********:**** ***::************:*: :*


C. maris         NHLRWDSLGEFLALAASLEHVAVTTGNARAQILADTLDAATGKFLDTNKSPSRKVGELDN 658
34H              NHLRWDSLGEFLALAASLEHVAISTGNAKAQVLADTLDSATGKFLDTNKSPSRRVGELDN 658
A.vinelandii     GYLRWDSLGEFLALAASLEHLGNAYKNPKALVLASTLDQATGKILDNNKSPARKVGEIDN 656
P. psychrophila  NFLRWDSLGEFLALAASLEHLGTTYNNPKALVLAKTLDQATGQFLDNNKSPSRKVGNIDN 656
P. putida KT2440 NFLRWDSLGEFLALAASLEHLGNTYDNPRAKVLANTLDQATGKFLDTNKSPSRKVGGIDN 657
P. aeruginosa    NYLRWDSLGEFLALAVSLEETGIKTGNAKAKLLGKALDEATGKLLDNNKSPSRKVGDIDN 656
34H_2            NHLRWDSLGEFLAISASLEDLGIKQDSAKAKLLAKTLDQATAELLENGKSPSRKTGELDN 656
M. tuberculosis  NHLRWDSLGEFLALGAGFEDIGIKTGNERAKLLGKTLDAAIGKLLDNDKSPSRKTGELDN 659
                 ..***********:...:*. .    . :* :*..:** * .::*:..***:*:.* :**

C. maris         RGSHFYLAMYWAQALAAQTTDTELQASFSSVAQALTKQEEKIVAELNAAQGPAIDLNGYY 718
34H              RGSHFYLAMYWAQAIAEQTTDTDLKESFTGVAQALTKQEEKIVAELNAAQGPAIDVNGYY 718
A.vinelandii     RGSHFYLALYWAQALAAQTEDKELQAQFTGIAKALTDNETKIVGELAAAQGKPVDIAGYY 716
P. psychrophila  RGSHFYLALYWAQALAAQTEDKELQAQFAPVAKAMAENEAKIVAELNAVQGKPVDIGGYY 716
P. putida KT2440 RGSHFYLTLYWAEALAAQTDDAALQARFAPLAKTLAENEATIVAELNAVQGKPADIGGYY 717
P. aeruginosa    RGSHFYLAMYWAQALAAQNEDAELKAHFAPLAKALTEQEATIVAELNAVQGKPAEIGGYY 716
34H_2            RGSHFYLALYWAKALAEQTEDTELQHHFAPLAQSLTENEAAIVSDLTTDQWHSVDIGGYY 716
M. tuberculosis  RGSQFYLAMYWAQELAAQTDDQQLAEHFASLADVLTKNEDVIVRELTEVQGEPVDIGGYY 719
                 ***:***::***: :* *. *  *   *: :*. ::.:*  ** :*   *    :: ***

C. maris         FADTKLAEKAMRPSETFNTILSALL- 743
34H              FADTQLTEKAMRPSETLNTILSALL- 743
A.vinelandii     HPNTDLTSKAIRPSATFNAALAPLA- 741
P. psychrophila  HADADKLSKVMRPSATLNAIIASLV- 741
P. putida KT2440 APDAELTAKVMRPSQTLNSAIAAL-- 741
P. aeruginosa    RSNPELTSKVMRPSATFNAAIDSLA- 741
34H_2            KADAKKIIEIMRPSKCFNEVLAAFK- 741
M. tuberculosis  APDSDMTTAVMRPSKTFNAALEAVQG 745
                   : .     :***  :*  :  .
```

**Fig. S5. Monomeric isocitrate dehydrogenase multiple alignment using Clustal W.** The amino acids that confer NADP^+^-coenzyme specificity and are responsible for metal (Mg^2+^, Mn^2+^) and substrate binding are indicated in shaded pink, light green and blue colors, respectively. The amino acid residues involved in the mesophilic properties of the *P. psychrophila* monomeric are indicated in bold green letters. The amino acid residues that are different from *C. maris*/*C. psychrerythrea* and *P. psychrophila* are shown in bold blue letters [36]. The proposed amino acids responsible for the thermal properties are shown in shaded dark green color [29]. The point mutations realized in this study (pET29-PP_4012mut) are indicated in shaded yellow color. * (asterisk) indicates positions, which have a single, fully conserved residue; : (colon) indicates conservation between groups of strongly similar properties - scoring > 0.5 in the Gonnet PAM 250 matrix; . (period) indicates conservation between groups of weakly similar properties - scoring =< 0.5 in the Gonnet PAM 250 matrix. The database accession numbers are for *C. maris* (BAA03134), *C. psychrerythraea* 34H (AAZ27772/AAZ27953), *P. psychrophila* (BAH80317), *P. aeruginosa* PAO1 (AAG06012), *P. putida* KT2440 (NP_746142), *M. tuberculosis* H37Rv (NP_214580), and *A. vinelandii* (D73443/BAA11169).

**Additional structural Information of PIDHs**

ICD, IDH lacks the AceK recognition segment, precluding its regulation by phosphorylation at Ser115 in *P. putida* [34,39,40]. This regulatory mechanism is supported by the presence of the *aceK* gene (PP_4565) in its genome. Here, *in silico* modeling of the AceK-ICD complex revealed that one domain of AceK interacts with the catalytic center of an ICD monomer, positioning the P-loop, where Ser115 is located, for its phosphorylation, likely leading to ICD inhibition (Fig. S6).

**Fig. S6.** **A.** Structural model of the homodimer of the *P. putida* KT2440 dimeric ICD protein. The active site of monomer A, NADP^+^-binding residues are represented in blue, isocitrate-binding amino acids in green, and metal ion binding residues in orange. The P-loop residue Ser115 is represented in yellow. **B.** Structural model of monomeric IDH from *P. putida* KT2440. Amino acids involved in NADP^+^ binding are shown in blue, those involved in metal ion binding are coloured in orange while those responsible for isocitrate binding are represented in green. C. Predicted complex between the dimeric ICD homodimer and the AceK (PP_4565) protein. The Ser115 residue, susceptible to phosphorylation by AceK, is highlighted in yellow. The PYMOL files may be found at the following link: https://saco.csic.es/s/Arrb3eLKRx4tyrB

**Fig. S7. Expression correlations of *ICDHYr* genes.** The figure displays correlations between both *ICDHYr* genes and the previously mentioned TCA targets using the putida PRECISE321 dataset from iModulonDB (<https://imodulondb.org>), which contains samples of 321 samples among 118 different conditions. Color refers to correlation groups of *idh* (A-D), *icd* (F-H) or the correlation test between them (E). The used correlation parameter was the Pearson correlation coefficient (R2), being the p value (p-Val) obtained for assuming as null hypothesis that the distributions underlying the samples are uncorrelated. Both parameters were computed though their *scipy* implementation.

**REFERENCES**

1. Howard L, Becker RR. Isolation and Some Properties of the Triphosphopyridine Nucleotide Isocitrate Dehydrogenase from *Bacillus stearothermophilus*. 245:9.

2. Singh SK, Matsuno K, LaPorte DC, Banaszak LJ. Crystal Structure of *Bacillus subtilis* Isocitrate Dehydrogenase at 1.55 Å: INSIGHTS INTO THE NATURE OF SUBSTRATE SPECIFICITY EXHIBITED BY *ESCHERICHIA COLI* ISOCITRATE DEHYDROGENASE KINASE/PHOSPHATASE. J Biol Chem. 2001;276:26154–63. https://doi.org/10.1074/jbc.M101191200

3. Singh SK, Miller SP, Dean A, Banaszak LJ, LaPorte DC. *Bacillus subtilis* Isocitrate Dehydrogenase: A SUBSTRATE ANALOGUE FOR *ESCHERICHIA COLI* ISOCITRATE DEHYDROGENASE KINASE/PHOSPHATASE. J Biol Chem. 2002;277:7567–73. https://doi.org/10.1074/jbc.M107908200

4. Huang SP, Cheng HM, Wang P, Zhu GP. Biochemical characterization and complete conversion of coenzyme specificity of isocitrate dehydrogenase from *bifidobacterium longum*. Int J Mol Sci. 2016;17. https://doi.org/10.3390/ijms17030296

5. Cozzone AJ, El-Mansi M. Control of Isocitrate Dehydrogenase Catalytic Activity by Protein Phosphorylation in *Escherichia coli*. J Mol Microbiol Biotechnol. 2005;9:132–46. https://doi.org/10.1159/000089642

6. Watanabe S, Yasutake Y, Tanaka I, Takada Y. Elucidation of stability determinants of cold-adapted monomeric isocitrate dehydrogenase from a psychrophilic bacterium, *Colwellia maris,* by construction of chimeric enzymes. Microbiology. 2005;151:1083–94. https://doi.org/10.1099/mic.0.27667-0

7. Romkina AY, Kiriukhin MY. Biochemical and molecular characterization of the isocitrate dehydrogenase with dual coenzyme specificity from the obligate methylotroph Methylobacillus Flagellatus. Permyakov EA, editor. PLOS ONE. 2017;12:e0176056. https://doi.org/10.1371/journal.pone.0176056

8. Wang P, Lv C, Zhu G. Novel Type II and Monomeric NAD+ Specific Isocitrate Dehydrogenases: Phylogenetic Affinity, Enzymatic Characterization, and Evolutionary Implication. Sci Rep. 2015;5:1–11. https://doi.org/10.1038/srep09150

9. Wang P, Song P, Jin M, Zhu G. Isocitrate Dehydrogenase from Streptococcus mutans: Biochemical Properties and Evaluation of a Putative Phosphorylation Site at Ser102. Tuma R, editor. PLoS ONE. 2013;8:e58918. https://doi.org/10.1371/journal.pone.0058918

10. Eguchi H, Wakagi T, Oshima T. A highly stable NADP-dependent isocitrate dehydrogenase from Thermus thermophilic HB8: purification and general properties. Biochim Biophys Acta BBA - Gen Subj. 1989;990:133–7. https://doi.org/10.1016/S0304-4165(89)80024-8

11. Wang P, Jin M, Zhu G. Biochemical and molecular characterization of NAD+-dependent isocitrate dehydrogenase from the ethanologenic bacterium Zymomonas mobilis. FEMS Microbiol Lett. 2012;327:134–41. https://doi.org/10.1111/j.1574-6968.2011.02467.x

12. Wang P, Wu Y, Liu J, Song P, Li S, Zhou X, et al. Crystal Structure of the Isocitrate Dehydrogenase 2 from Acinetobacter baumannii (AbIDH2) Reveals a Novel Dimeric Structure with Two Monomeric-IDH-Like Subunits. Int J Mol Sci. 2018;19:1131. https://doi.org/10.3390/ijms19041131

13. Reeves HC, O’Neil S, Weitzman PDJ. Modulation of isocitrate dehydrogenase activity in *Acinetobacter calcoaceticus* by acetate. FEBS Lett. 1983;163:265–8. https://doi.org/10.1016/0014-5793(83)80832-1

14. Reeves HC, O’Neil S, Weitzman PDJ. Changes in NADP-isocitrate dehydrogenase isoenzyme levels in *Acinetobacter calcoaceticus* in response to acetate. FEMS Microbiol Lett. 1986;35:229–32. https://doi.org/10.1111/j.1574-6968.1986.tb01533.x

15. Yasutake Y, Watanabe S, Yao M, Takada Y, Fukunaga N, Tanaka I. Structure of the Monomeric Isocitrate Dehydrogenase. Structure. 2002;10:1637–48. https://doi.org/10.1016/S0969-2126(02)00904-8

16. Yasutake Y, Watanabe S, Yao M, Takada Y, Fukunaga N, Tanaka I. Crystal Structure of the Monomeric Isocitrate Dehydrogenase in the Presence of NADP ^+^: INSIGHT INTO THE COFACTOR RECOGNITION, CATALYSIS, AND EVOLUTION. J Biol Chem. 2003;278:36897–904. https://doi.org/10.1074/jbc.M304091200

17. Kanao T, Kawamura M, Fukui T, Atomi H, Imanaka T. Characterization of isocitrate dehydrogenase from the green sulfur bacterium chlorobium limicola: A carbon dioxide-fixing enzyme in the reductive tricarboxylic acid cycle. Eur J Biochem. 2002;269:1926–31. https://doi.org/10.1046/j.1432-1033.2002.02849.x

18. Eikmanns BJ, Rittmann D, Sahm H. Cloning, sequence analysis, expression, and inactivation of the Corynebacterium glutamicum icd gene encoding isocitrate dehydrogenase and biochemical characterization of the enzyme. J Bacteriol. 1995;177:774–82. https://doi.org/10.1128/jb.177.3.774-782.1995

19. Chen R, Yang H. A highly specific monomeric isocitrate dehydrogenase from Corynebacterium glutamicum. Arch Biochem Biophys. 2000; https://doi.org/10.1006/abbi.2000.2082

20. Steen IH, Madsen MS, Birkeland N-K, Lien T. Purification and characterization of a monomeric isocitrate dehydrogenase from the sulfate-reducing bacterium *Desulfobacter vibrioformis* and demonstration of the presence of a monomeric enzyme in other bacteria. FEMS Microbiol Lett. 1998;160:75–9. https://doi.org/10.1111/j.1574-6968.1998.tb12893.x

21. Hirota R, Tsubouchi K, Takada Y. NADP+-dependent isocitrate dehydrogenase from a psychrophilic bacterium, Psychromonas marina. Extremophiles. 2017;21:711–21. https://doi.org/10.1007/s00792-017-0936-0

22. McDermott TR, Kahn ML. Cloning and mutagenesis of the Rhizobium meliloti isocitrate dehydrogenase gene. J Bacteriol. 1992;174:4790–7. https://doi.org/10.1128/jb.174.14.4790-4797.1992

23. Zhang B, Wang B, Wang P, Cao Z, Huang E, Hao J, et al. Enzymatic characterization of a monomeric isocitrate dehydrogenase from Streptomyces lividans TK54. Biochimie. 2009;91:1405–10. https://doi.org/10.1016/j.biochi.2009.07.011

24. Fukunaga N, Sasaki S, Minowa O, Mizuno Y, Shiokawa H, Science I. Isozymes Psychrophilic of Isocitrate Bacterium , Dehydrogenase Vibrio Strain an Obligately and Modulation of Activities by Growth Conditions and quaternary homogeneous state from an obligately psychrophilic marine chromatography was an efficient procedure. 1987;102:1489–98.

25. Ochiai T, Fukunaga N, Sasaki S. Purification and some properties of two NADP+-specific isocitrate dehydrogenases from an obligately psychrophilic marine bacterium, Vibrio sp., strain ABE-1. J Biochem (Tokyo). 1979;86:377–84. https://doi.org/10.1093/oxfordjournals.jbchem.a132536

26. Ishii A, Suzuki M, Sahara T, Takada Y, Sasaki S, Fukunaga N. Genes encoding two isocitrate dehydrogenase isozymes of a psychrophilic bacterium , Vibrio Genes Encoding Two Isocitrate Dehydrogenase Isozymes of a Psychrophilic Bacterium , Vibrio sp . Strain ABE-1. J Bacteriol. 1993;175:6873–80.

27. Suzuki M, Sahara T, Tsuruha JI, Takada Y, Fukunaga N. Differential expression in Escherichia coli of the Vibrio sp. strain ABE-1 icdI and icdII genes encoding structurally different isocitrate dehydrogenase isozymes. J Bacteriol. 1995;177:2138–42. https://doi.org/10.1128/jb.177.8.2138-2142.1995

28. Yasuda W, Kobayashi M, Takada Y. Analysis of amino acid residues involved in cold activity of monomeric isocitrate dehydrogenase from psychrophilic bacteria, Colwellia maris and Colwellia psychrerythraea. J Biosci Bioeng. 2013;116:567–72. https://doi.org/10.1016/j.jbiosc.2013.05.012

29. Kobayashi M, Takada Y. Effects of the combined substitutions of amino acid residues on thermal properties of cold-adapted monomeric isocitrate dehydrogenases from psychrophilic bacteria. Extremophiles. 2014;18:755–62. https://doi.org/10.1007/s00792-014-0656-7

30. Maki S, Yoneta M, Takada Y. Two isocitrate dehydrogenases from a psychrophilic bacterium, Colwellia psychrerythraea. Extremophiles. 2006;10:237–49. https://doi.org/10.1007/s00792-005-0493-9

31. Suzuki K, Takada YT. Characterization of NADP+-dependent isocitrate dehydrogenase isozymes from a psychrophilic bacterium, Colwellia psychrerythraea strain 34H. Biosci Biotechnol Biochem. 2016;80:1492–8. https://doi.org/10.1080/09168451.2016.1165602

32. Wang Z-X, BrÃ¤mer C, SteinbÃ¼chel A. Two phenotypically compensating isocitrate dehydrogenases in *Ralstonia eutropha*. FEMS Microbiol Lett. 2003;227:9–16. https://doi.org/10.1016/S0378-1097(03)00612-8

33. Murima P, Zimmermann M, Chopra T, Pojer F, Fonti G, Dal Peraro M, et al. A rheostat mechanism governs the bifurcation of carbon flux in mycobacteria. Nat Commun;7. https://doi.org/10.1038/ncomms12527

34. Crousilles A, Dolan SK, Brear P, Chirgadze DY, Welch M. Gluconeogenic precursor availability regulates flux through the glyoxylate shunt in \textlessi\textgreaterPseudomonas aeruginosa\textless/i\textgreater. J Biol Chem. 2018; https://doi.org/10.1074/jbc.RA118.004514

35. Roy SO, Packard TT. NADP-Isocitrate Dehydrogenase from Pseudomonas nautica: Kinetic Constant Determination and Carbon Limitation Effects on the Pool of Intracellular Substrates. APPL Env MICROBIOL. 1998;64:7.

36. Matsuo S, Shirai H, Takada Y. Isocitrate dehydrogenase isozymes from a psychrotrophic bacterium, Pseudomonas psychrophila. Arch Microbiol. 2010;192:639–50. https://doi.org/10.1007/s00203-010-0595-3

37. Lv P, Tang W, Wang P, Cao Z, Zhu G. Enzymatic characterization and functional implication of two structurally different isocitrate dehydrogenases from *Xylella fastidiosa*: Two Isocitrate Dehydrogenases from *Xylella*. Biotechnol Appl Biochem. 2018;65:230–7. https://doi.org/10.1002/bab.1560

38. Manoli M-T, Gargantilla-Becerra Á, Del Cerro Sánchez C, Rivero-Buceta V, Prieto MA, Nogales J. A model-driven approach to upcycling recalcitrant feedstocks in Pseudomonas putida by decoupling PHA production from nutrient limitation. Cell Rep. 2024;43:113979. https://doi.org/10.1016/j.celrep.2024.113979

39. Walsh K, Koshland DE. Branch point control by the phosphorylation state of isocitrate dehydrogenase. A quantitative examination of fluxes during a regulatory transition. J Biol Chem. 1985;260:8430–7.

40. Dean AM, Lee MH, Koshland DE. Phosphorylation inactivates Escherichia coli isocitrate dehydrogenase by preventing isocitrate binding. J Biol Chem. 1989;264:20482–6.
